# Low frequency vibrating magnetic field-triggered magnetic microspheres with a nanoflagellum-like surface for cancer therapy

**DOI:** 10.1186/s12951-022-01521-7

**Published:** 2022-07-06

**Authors:** Yuliang Guo, Wenxuan Yang, Guangjin Pu, Chunjiao Zhu, Yifan Zhu, Ji Li, Yuqiao Huang, Bo Wang, Maoquan Chu

**Affiliations:** 1 Research Center for Translational Medicine at Shanghai East Hospital, School of Life Sciences and Technology, Tongji University, Shanghai, 200092 People’s Republic of China; 2 School of Physics Science and Engineering, Tongji University, Shanghai, 200092 People’s Republic of China

**Keywords:** Magnetic microspheres, Flagellum-like surface, Magneto-mechanical force, Reactive oxygen species, Cancer therapy

## Abstract

**Background:**

The magneto-mechanical force killing cancer cells is an interesting and important strategy for cancer therapy.

**Results:**

Novel magnetic microspheres composed of a Fe_3_O_4_ nanocore, a bovine serum albumin (BSA) matrix, and a rod-like SiO_2_ nanoshell, which had flagellum-like surface for force-mediated cancer therapy were developed. One such magnetic microsphere (Fe_3_O_4_/BSA/rSiO_2_) at a cancer cell (not leave the cell surface) under a low frequency vibrating magnetic field (VMF) could generate 6.17 pN force. Interestingly, this force could induce cancer cell to generate reactive oxygen species (ROS). The force and force-induced ROS could kill cancer cells. The cell killing efficiency of Fe_3_O_4_/BSA/rSiO_2_ exposed to a VMF was enhanced with increasing silica nanorod length, and the microspheres with straight nanorods exhibited stronger cell killing ability than those with curled nanorods. Fe_3_O_4_/BSA/rSiO_2_ triggered by a VMF could efficiently inhibit mouse tumor growth, while these microspheres without a VMF had no significant effect on the cell cycle distribution, cell viability, tumor growth, and mouse health*.*

**Conclusions:**

These microspheres with unique morphological characteristics under VMF have great potential that can provide a new platform for treating solid tumors at superficial positions whether with hypoxia regions or multidrug resistance.

**Supplementary Information:**

The online version contains supplementary material available at 10.1186/s12951-022-01521-7.

## Background

Magnetic particles being remotely controlled by a magnetic field can mechanically move, so they are ideal materials to deliver a force to cells and tissues under exposure to a magnetic field. It has been demonstrated that the magnetic particle-mediated magneto-mechanical force of only a few tens of piconewtons acting on a cell membrane can induce cell death [1]. This cancer therapy method has attracted much attention because of its effectiveness, safety, easy operation, reusability, and low cost. Compared with alternating magnetic field-induced magnetic particle-mediated tumor hyperthermia [2], the equipment for producing a magneto-mechanical force does not require high electric voltage and strong current. A magnetic field with very small strength and low frequency (e.g., 90 Oe, 20 Hz) is sufficient to drive a magnetic particle to move in various ways, such as twisting [1], linear reciprocating vibration [3–5], rotation [6–12], and more complex motions [13–19].

Both nano- [4–8, 10–16] and microscale [1, 3, 5, 8, 17–19] magnetic particles have been investigated for damaging cancer cells via the magneto-mechanical force. Large or aggregated magnetic particles triggered by a dynamic magnetic field can easily damage cancer cells through generating large holes in the cell membrane owing to the large volume of particles. The large holes in the cell membrane provide many opportunities for cellular content release from the cell, which results in rapid cell death.

Magnetic particles with rough surfaces may exhibit strong ability for damaging the cancer cell membrane through the magneto-mechanical force. In this study, magnetic particles with sharp surfaces were fabricated by modifying the particles with silica rods. Silica nanoparticles have been used in clinic as silica materials usually exhibit excellent biocompatibility [20–25]. The morphology and length to diameter ratio of silica rods can be controlled by adjusting the reaction conditions. However, silica rods grown on magnetic particle surfaces and magnetic particle–silica rod core/shell composites for cancer therapy triggered by a magnetic field have not been reported.

**Scheme 1:**
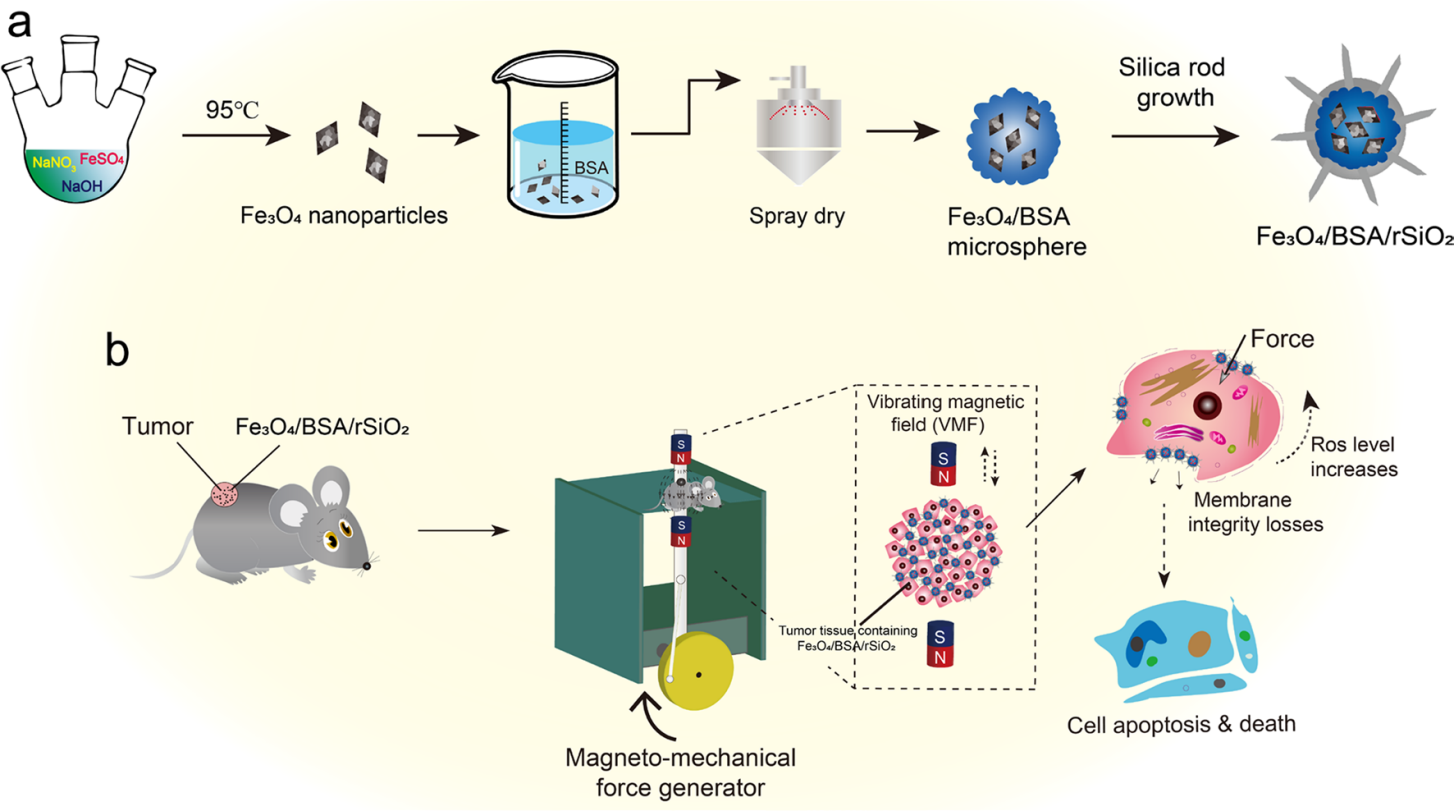
Schematic illustration of Fe_3_O_4_/BSA microspheres coated with rod-like SiO_2_ (Fe_3_O_4_/BSA/rSiO_2_) for cancer therapy. **a** Synthesis of the Fe_3_O_4_/BSA/rSiO_2_ magnetic microspheres. **b** Magneto-mechanical force and ROS generated by the microspheres exposed to a VMF for killing cancer cells and inhibiting tumor growth

In this study, a bovine serum albumin (BSA) aqueous solution containing superparamagnetic Fe_3_O_4_ nanoparticles was spray dried, followed by protein denaturation and coating with silica nanorods with different lengths through heterogeneous chemical reaction in aqueous solution (Scheme 1a). The silica nanorods grew on the magnetic Fe_3_O_4_/BSA microsphere surface because the microspheres provided nucleation sites for growth of silica rods. Numerous silica rods grown on the microspheres made the microspheres exhibit a flagellum-like surface and significantly enhanced the sharp features, which is beneficial for damaging cancer cells through mechanical force. A vibrating magnetic field (VMF) with low frequency triggered the magnetic microspheres to efficiently kill cancer cells, and this cell killing efficiency was dependent on the silica rod length, VMF frequency, exposure time, and microsphere concentration. A large number of large holes were clearly observed on the cancer cells after the cells were treated with the microspheres with sharp surfaces under a VMF. The cancer cells were killed and the tumor growth in the mouse body was inhibited by both the magneto-mechanical force and force-induced intracellular reactive oxygen species (ROS) (Scheme 1b). ROS exhibits high toxic to cells and tissues. Such force-induced ROS for killing cancer cells has been defined as force-dynamic therapy (FDT) [26].

The strategy reported in this study may be suitable for inhibiting other solid tumor growth besides the laryngeal neoplasm investigated. Because Fe_3_O_4_ nanoparticles, BSA, and SiO_2_ all are biocompatible materials and the applied magnetic field is at low frequency, the as-developed magnetic microspheres coated with silica rods and the method for inhibiting tumor growth through the magneto-mechanical force and ROS show potential for clinical application.

## Materials and methods

Materials, cell line and animals were described in the Additional file 1.

### Preparation of the Fe_3_O_4_/BSA hybridized microspheres

Concentrated H_2_SO_4_ (20 μL) and FeSO_4_·7H_2_O (5.56 g) were added to dissolved oxygen-free distilled water. The mixture was then slowly added to NaOH aqueous solution (100 mL, 16 mg/mL) with vigorous mechanical stirring under nitrogen flow. The reaction suspension was heated to 90 °C and kept at this temperature for 2 h. The suspension was then naturally cooled to room temperature. The suspension in both the heating and cooling stages was protected by nitrogen. The precipitates were collected by magnetic separation and washed with distilled water and absolute ethanol three times. The washed black precipitates (i.e., Fe_3_O_4_ nanoparticles) were freeze dried and then stored in a refrigerator at 4 °C.

The Fe_3_O_4_ nanoparticles (3.5 g) were dispersed in BSA aqueous solution (500 mL, 9 mg/mL). The suspension was then spray dried using a spray dryer (6000Y, Qiqian Electronic Technology, Shanghai, China). The inlet temperature, wind speed, and feed rate were maintained at 70 °C, 20 m/s, and 5 mL/min, respectively. After feeding, the inlet temperature was increased to 100 °C and maintained at this temperature for about 5 min. The powder was then collected and heated to 180 °C in a quartz tube furnace (SLG1100-60, Shanghai Shengli Test Instruments Co. Ltd., Shanghai, China) and maintained at this temperature for 2 h under nitrogen flow. After cooling to room temperature, the powder was immersed in tetrahydrofuran solution for 48 h. The precipitates were then collected by magnetic separation and washed with absolute ethanol at least five times. The final precipitates were then washed with distilled water, followed by freeze drying. The dried powder (i.e., Fe_3_O_4_/BSA microspheres) was stored in a refrigerator at 4 °C.

### Synthesis of Fe_3_O_4_/BSA/rSiO_2_ with different length SiO_2_ nanorods

For synthesis of silica rod-coated Fe_3_O_4_/BSA, PVP powder (5 g) was dissolved in a mixture of *n*-pentanol (50 mL) and absolute ethanol (5 mL), followed by addition of citric acid sodium aqueous solution (0.335 mL, 0.18 M). Distilled water, Fe_3_O_4_/BSA microsphere powder (5 mg), and TEOS (0.5 mL) were then sequentially added to the above mixture solution under sonication treatment. The volume of distilled water was 2, 1.3, 0.7, and 0.35 mL for synthesis of short, middle, and long silica rods, and curled silicon rods, respectively. After the microspheres were well dispersed in the above solution, ammonia (1 mL, 28%) was slowly added to the suspension under gentle stirring. After about 20 h, the precipitates were collected by magnetic separation and washed with absolute ethanol about 15 times. The precipitates were then washed with distilled water, dispersed in absolute ethanol, and stored in a refrigerator at 4 °C. Before use, the samples were centrifuged and washed with water to remove ethanol.

### Evaluation of the morphology of cancer cells damaged by Fe_3_O_4_/BSA/rSiO_2_ triggered by a VMF

The Tu212 cells (~ 8 × 10^4^) were seeded on cover slips (2 cm × 2 cm) in culture dishes. After culture for ~ 10 h, the RPMI-1640 culture medium was removed and the cells were washed with PBS. At the same time, Fe_3_O_4_/BSA/rSiO_2_ with long silica rods were dispersed in serum-free medium and immediately added to the cells (2 mg/mL for each sample, 100 μL for each well), followed by VMF exposure for 0.5, 1, and 1.5 h. The VMF strength and frequency were maintained at 400 mT and 2 Hz. The critical parts of the VMF equipment are composed of two permanent magnets and a rotating motor. The magnets exhibit straight-line reciprocating motion triggered by the motor, which has been reported in our previous work [3]. After exposure, the cells were fixed with 2.5% glutaraldehyde at 4 °C for 2 h, followed by PBS washing, dehydration using a gradient series of acetone (30%, 50%, 70%, 90%, and 100%), and SEM observation. The cells incubated with the same Fe_3_O_4_/BSA/rSiO_2_ microspheres without VMF exposure and the cells alone were also characterized by SEM.

### Determination of the intracellular ROS level generated by Fe_3_O_4_/BSA/rSiO_2_ triggered by a VMF

To determine the intracellular ROS level, Tu212 cells (~ 1 × 10^4^) were cultured in a 96-well plate. After the cells were washed with PBS, serum-free medium-dispersed Fe_3_O_4_/BSA/rSiO_2_ with long silica rods (0.2, 0.5, and 1 mg/mL) was added to the cells, followed by exposure to a VMF for 1 h. The VMF strength and frequency were maintained at 400 mT and 2 Hz. Tu212 cells incubated with Fe_3_O_4_/BSA/rSiO_2_ without VMF exposure, Tu212 cells alone with VMF exposure for 1 h, and Tu212 cells without VMF exposure were used as controls. After VMF exposure, RPMI-1640-dissolved DCFH-DA reagent (100 μL, DCFH-DA/medium = 1/1000) was added to the cells and incubated at 37 °C for 20 min, followed by PBS washing. PBS (100 μL per well) was added for measurement of the fluorescence intensity (FI) at 525 nm (excitation 488 nm) using an universal microplate spectrophotometer (SpectraMax M5, Molecular Devices, USA). The ratio of FI of the treatment group to that of the Tu212 cells alone was defined as the ROS level. Each experiment was repeated five times.

### Measurement of the cell killing efficiency of Fe_3_O_4_/BSA/rSiO_2_ triggered by a VMF

To measure the cell killing efficiency, Tu212 cells were cultured in a 96-well plate and washed with PBS using the same method described above. Serum-free medium-dispersed Fe_3_O_4_/BSA and Fe_3_O_4_/BSA/rSiO_2_ with different length silica rods (1 mg/mL for each sample, 100 μL for each well) were added to the cells. After incubation at 37 °C for 1 h, the cells were exposed to a VMF (2 Hz) for 1 h. After exposure, the cells were continuously cultured at 37 °C for 2 h. The cell viabilities were qualitatively detected using Hoechst33342/PI double reagent, and the cells were imaged with an inverted fluorescent microscope. The cell viabilities were also quantitatively determined using CellTiter-Glo® reagent. For quantitative determination, the methods of cell culturing and VMF exposure were the same as above, but each experiment was repeated five times.

The cell viabilities in the following three cases were also quantitatively determined five times: Tu212 cells incubated with Fe_3_O_4_/BSA/rSiO_2_ with three other concentrations (0.2, 0.5, and 2 mg/mL) and then exposed to the same VMF, Tu212 cells incubated with Fe_3_O_4_/BSA/**L**rSiO_2_ (1 mg/mL) and then exposed to the VMF for 0.5 and 1.5 h, and Tu212 cells incubated with Fe_3_O_4_/BSA/rSiO_2_ (1 mg/mL) and then exposed to the VMF with frequency of 0.5, 1, 3, and 4 Hz for 1 h. To further evaluate the cell killing efficiency, LDH release was also measured, which is described in the Additional file 1.

### Study of in vivo tumor inhibition of Fe_3_O_4_/BSA/rSiO_2_ triggered by a VMF

To investigate in vivo tumor inhibition, Tu212 cells were digested and washed three times with PBS, and then mixed with PBS-dispersed Fe_3_O_4_/BSA/rSiO_2_ under ice bath conditions. The cell concentration was 5 × 10^6^ cells/mL and the concentration of Fe_3_O_4_/BSA/rSiO_2_ was 5 or 15 mg/mL. The cells were then subcutaneously injected into sixteen nude mice near the right forelimb (50 μL for each mouse, *n* = 8 for each concentration). Twelve hours post-injection, the injection sites on eight mice (*n* = 4 for each concentration) were exposed to a VMF (400 mT, 2 Hz) for 1 h per day, and the other eight mice were not exposed to the VMF. The mice (*n* = 4) subcutaneously injected with PBS-dispersed Tu212 cells alone without VMF exposure were used as controls. The VMF was stopped after the mice were exposed for 21 days. The mice were normally fed for 21 days. The tumor sizes were measured and photos of the mice were taken every 7 days. The tumor volume was calculated using the following equation: tumor volume = (length × width × width)/2. At 42 days post-injection, the mice were weighted and then sacrificed. The mouse tumors and main organs, including the heart, liver, spleen, lungs, and kidneys, were resected and weighted, followed by fixation in Bouin’s solution and embedding in paraffin wax using the standard protocols of tissue fixation and embedding. The embedded tissues were then cut into ultrathin sections using an ultramicrotome (Leica RM2126RT, Germany), followed by staining with hematoxylin–eosin (H&E) stain reagent. The sections were then analyzed with an upright fluorescence microscope (Axio Imager. M2, Carl Zeiss, Germany).

To evaluate the tumor temperature increase, PBS-dispersed Fe_3_O_4_/BSA/rSiO_2_ (10 mg/mL, 50 *μ*L) was injected into a mouse tumor (~ 32 mm^3^, grow from Tu212 cells). About 10 h later, the tumor was exposed to the VMF (400 mT, 2 Hz) for 1 h. During exposure, the tumor temperature was recorded using an infrared thermal imager (Ti29, Fluke Corporation, Everett, WA, USA). The temperature of PBS-dispersed Fe_3_O_4_/BSA/rSiO_2_ (10 mg/mL, 50 *μ*L) exposed to the same VMF was also recorded. All the above animal experiments were performed in accordance with the University of Tongji Institutional Animal Care and Use Committee Guidelines (No: TJAB05820102).

## Results and discussion

### Characterization of Fe_3_O_4_/BSA and Fe_3_O_4_/BSA/rSiO_2_

**Fig. 1:**
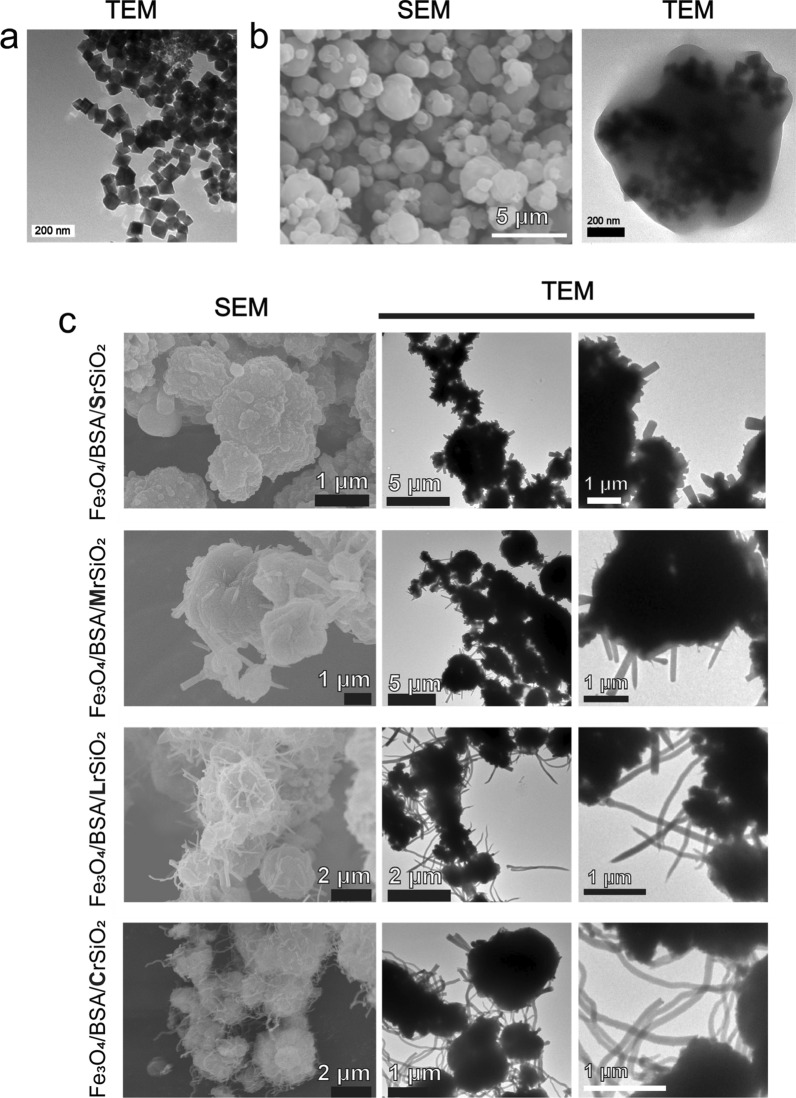
Morphologies of the samples. **a** Transmission electron microscopy (TEM) image of the Fe_3_O_4_ nanoparticles. **b** Scanning electron microscopy (SEM, left) and TEM (right) images of the BSA-coated Fe_3_O_4_ microspheres (i.e., Fe_3_O_4_/BSA). **c** SEM and TEM images of Fe_3_O_4_/BSA/rSiO_2_ microspheres with different length silica nanorods. The TEM images in the right panels are the microspheres at high magnification

Cubic superparamagnetic Fe_3_O_4_ nanoparticles with high saturation magnetization (76.25 emu/g) (Fig. 1a and Additional file 1: Figs. S1, S2) were synthesized. BSA microspheres containing Fe_3_O_4_ cores (i.e., Fe_3_O_4_/BSA) were then formed by spray drying followed by denaturation of BSA. In the obtained magnetic microspheres (Fig. 1b, left), a large number of Fe_3_O_4_ nanoparticles can be clearly observed in the internal space (Fig. 1b, right).

SiO_2_ nanorods could grow on the surfaces of the Fe_3_O_4_/BSA microspheres. The obtained Fe_3_O_4_/BSA/rSiO_2_ microspheres had a flagellum-like surface (Fig. 1c). The length of the silica nanorods could be tailored by adjusting the water volume in the reaction system (see methods). For example, when the volume of water added to the reaction precursor was changed from 2 to 1.3, 0.7, and 0.35 mL, silica nanorods with lengths of 305.3 ± 118.0 nm, 873.7 ± 178.2 nm, 1691.3 ± 302.2 nm, and curled silicon (more than 1700 nm) grew on the microsphere surfaces (Additional file 1: Fig. S3). In the following sections, these microspheres are referred to as Fe_3_O_4_/BSA/**S**rSiO_2_, Fe_3_O_4_/BSA/**M**rSiO_2_, Fe_3_O_4_/BSA/**L**rSiO_2_, and Fe_3_O_4_/BSA/**C**rSiO_2_, where the letters **S**, **M**,** L** and **C** indicate short, medium, long, and curled, respectively. The sizes of Fe_3_O_4_/BSA and Fe_3_O_4_/BSA/rSiO_2_ mainly ranged approximately from 0.5 to 2.5 μm and 1 to 4 μm, respectively.

**Fig. 2:**
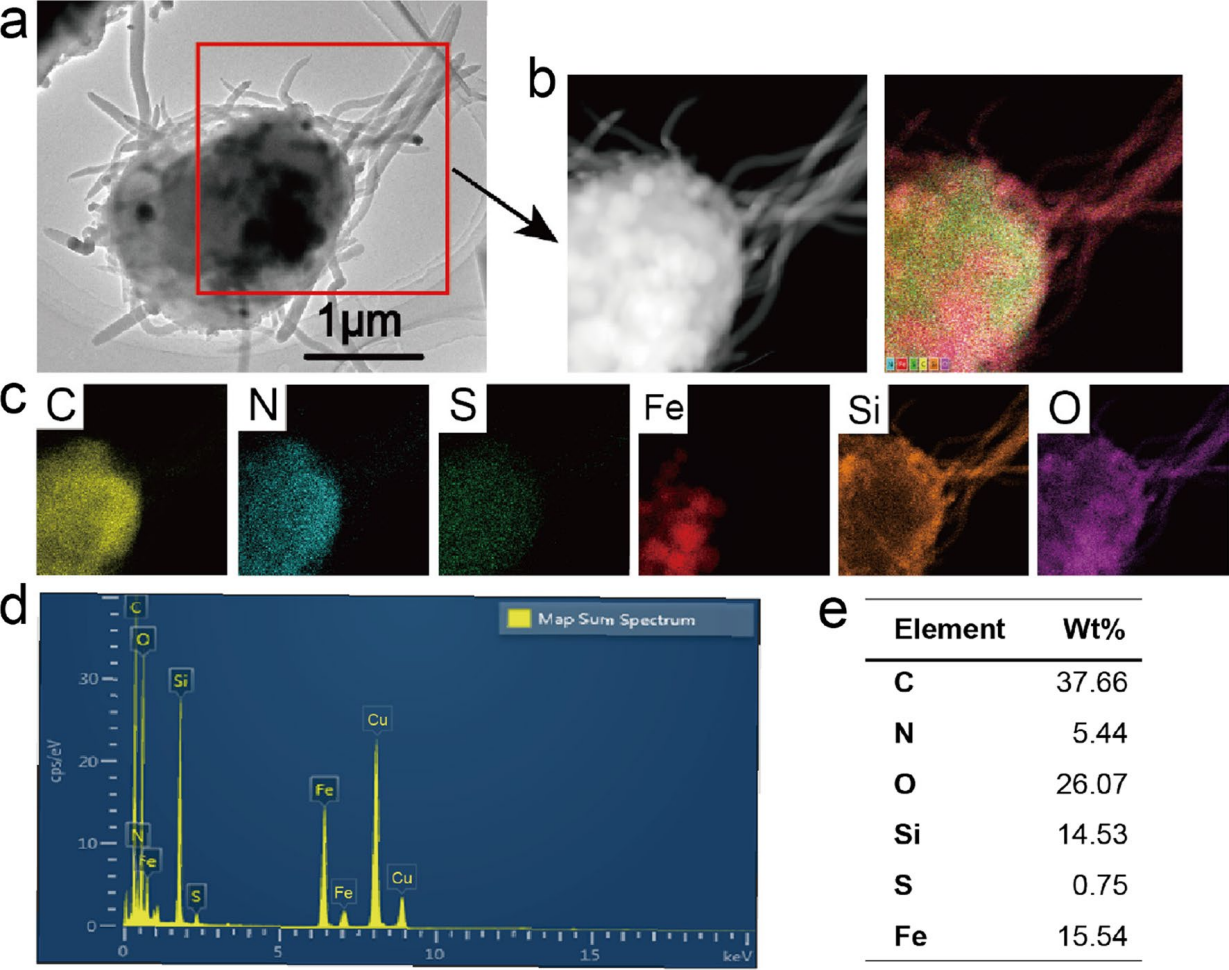
HRTEM elemental mapping and elemental component analysis of the Fe_3_O_4_/BSA/**L**rSiO_2_ microspheres. **a** HRTEM image. **b** Dark-field HRTEM image of the part of the microsphere shown in **a** and the corresponding energy dispersive X-ray spectroscopy (EDX) elemental mapping results (overlay image of the different elements). **c** EDX elemental mapping results of the sample shown in **b** based on the following elements: Fe, O, Si, C, N, and S. **d** EDS of the whole sample and **e** the corresponding percentage mass of each element (note: the peaks at ~ 8 and 9 keV are assigned to the element Cu of copper mesh)

High-resolution transmission electron microscopy (HRTEM) images clearly showed that Fe_3_O_4_/BSA/**L**rSiO_2_ contained numerous “dark” nanocores (Fig. 2a). These cores were iron oxide nanoparticles, because HRTEM elemental mapping revealed that Fe_3_O_4_/BSA/**L**rSiO_2_ contained C, O, Fe, Si, N, and S. The Fe atoms were located inside the microspheres and the microspheres were capped with a silica shell with a sharp surface (Fig. 2b and c). Energy-dispersive spectroscopy (EDS) of the whole sample confirmed that the Fe_3_O_4_/BSA/**L**rSiO_2_ microspheres were composed of the above six elements (Fig. 2d) and the mass ratio of Fe atoms was 15.54% (Fig. 2e). The mass of Fe atoms in the final microspheres can be easily adjusted by changing the concentration of Fe_3_O_4_ nanoparticles in the precursor solution before spray drying.

**Fig. 3:**
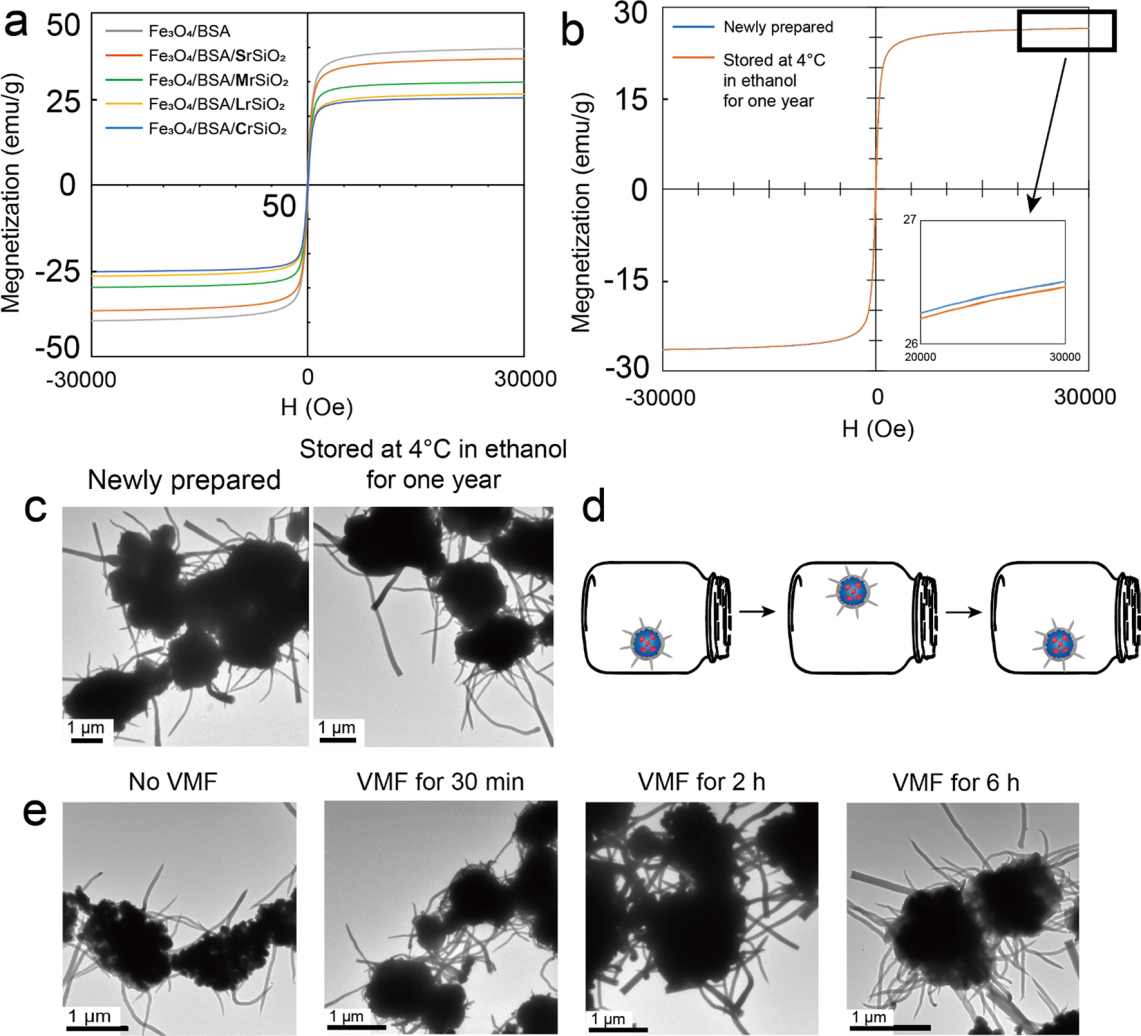
Stabilities of the magnetism and geometric structure of the Fe_3_O_4_/BSA/rSiO_2_ microspheres. **a** Magnetization hysteresis of Fe_3_O_4_/BSA and Fe_3_O_4_/BSA/rSiO_2_ with four types of silica rods. **b** Magnetization hysteresis and **c** morphology of Fe_3_O_4_/BSA/LrSiO_2_ before and after storage in absolute ethanol for 1 year. **d** Schematic illustration of Fe_3_O_4_/BSA/rSiO_2_ vibrating in a glass bottle under a VMF (a video was shown in the Additional file 2). **e** Morphology of Fe_3_O_4_/BSA/**L**rSiO_2_ before and after the exposure to a VMF for different time

The magnetic hysteresis loops of Fe_3_O_4_/BSA and Fe_3_O_4_/BSA/rSiO_2_ with four types of silica rods showed that all the samples maintained superparamagnetic character (the coercive force was close to zero) (Fig. 3a). The saturation magnetization of Fe_3_O_4_/BSA was 39.5 emu/g. It decreased after Fe_3_O_4_/BSA was coated with a silica shell, and it also decreased with increasing silica rod length, which is because SiO_2_ is a non-magnetic material (Fig. 3a). Fe_3_O_4_/BSA/rSiO_2_ could be well dispersed in aqueous solution, which may be owing to that both SiO_2_ and BSA are hydrophilic materials. For example, when Fe_3_O_4_/BSA/**L**rSiO_2_ were dispersed in PBS or RPMI-1640 culture medium, no precipitates could be observed at the bottom of tubes after being stored at room temperature for at least 5 min (Additional file 1: Fig. S4). After storing in absolute ethanol at 4 °C for 1 year, both the magnetism and morphology of Fe_3_O_4_/BSA/rSiO_2_ were almost unchanged. For example, for Fe_3_O_4_/BSA/**L**rSiO_2_, the saturation magnetization 1 year post-synthesis was close to 26 emu/g, which was similar to that of the as-synthesized sample (Fig. 3b). The silica rods on the Fe_3_O_4_/BSA microsphere surface and the Fe_3_O_4_/BSA microspheres did not obviously change in shape after 1 year storage (Fig. 3c), which showed their high stability during storage.

Owing to the magnetism, Fe_3_O_4_/BSA/rSiO_2_ exposed to a low frequency VMF (2 Hz) vigorously vibrated with vibration of the magnetic field (Fig. 3d and a video in the Additional file 2), which could be clearly observed directly. Furthermore, the morphology of the Fe_3_O_4_/BSA/rSiO_2_ microspheres did not obviously change after the microspheres were continuously exposed to a VMF for 6 h (Fig. 3e). These results indicated that Fe_3_O_4_/BSA/rSiO_2_ can be treated with a VMF for a long time, and such vibrating microspheres with sharp surfaces under a VMF may act as small weapons to fight cancer cells.

### Cytotoxicity of Fe_3_O_4_/BSA/rSiO_2_

#### *Effect of Fe*_*3*_*O*_*4*_*/BSA/rSiO*_*2*_* on the cell cycle distribution*

**Fig. 4:**
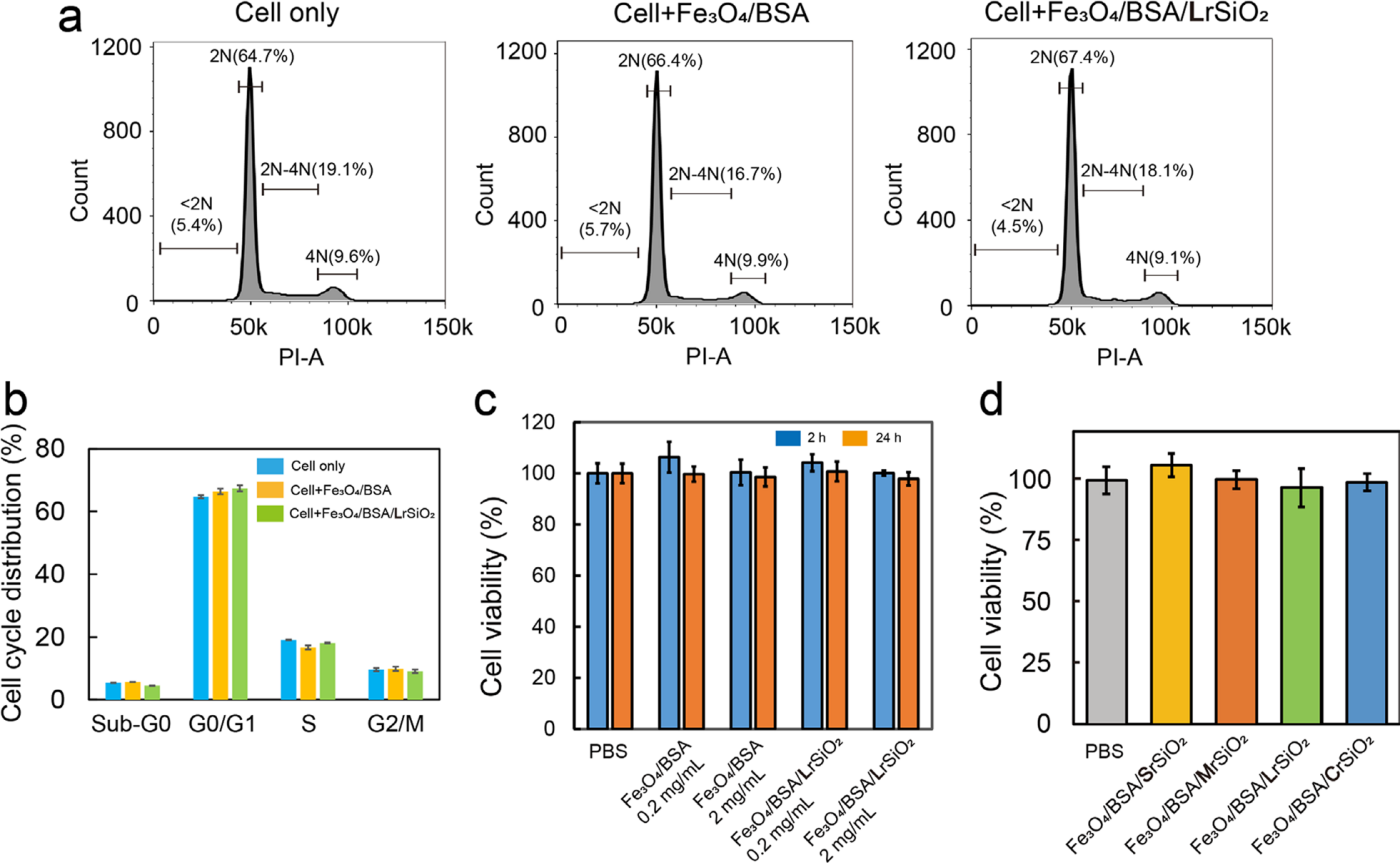
Cytotoxicity of Fe_3_O_4_/BSA and Fe_3_O_4_/BSA/rSiO_2_ with different length silica nanorods. Fe_3_O_4_/BSA/rSiO_2_ includes Fe_3_O_4_/BSA/SrSiO_2_, Fe_3_O_4_/BSA/MrSiO_2_, Fe_3_O_4_/BSA/LrSiO_2_, and Fe_3_O_4_/BSA/CrSiO_2_. **a** Representative images of the cell cycle after Tu212 cells were incubated with Fe_3_O_4_/BSA or Fe_3_O_4_/BSA/**L**rSiO_2_ (2 mg/mL for each sample) for 48 h. **b** Histograms of the cell cycle distribution. **c** Cell viability of Tu212 cells after being incubated with Fe_3_O_4_/BSA or Fe_3_O_4_/BSA/**L**rSiO_2_ under different conditions (sample concentrations: 0.2 and 2 mg/mL; incubation time: 2 and 24 h). **d** Comparison of the cell viability between PBS and the four types of Fe_3_O_4_/BSA/rSiO_2_ (sample concentration: 2 mg/mL; incubation time: 24 h)

After Tu212 cells were incubated with 0.5 mg/mL of Fe_3_O_4_/BSA or Fe_3_O_4_/BSA/**L**rSiO_2_ for 48 h, the populations of the cells in the G0/G1, S, and G2/M phases were all similar to those of the untreated cells (Fig. 4a and b). The cell cycle distribution was not significantly changed by the existence of Fe_3_O_4_/BSA or Fe_3_O_4_/BSA/**L**rSiO_2_, indicating that both materials have no influence to the cell life activities.

#### *Effect of Fe*_*3*_*O*_*4*_*/BSA/rSiO*_*2*_* on cell viability*

When Tu212 cells were incubated with Fe_3_O_4_/BSA or Fe_3_O_4_/BSA/**L**rSiO_2_ (0.2 or 2 mg/mL) for either 2 or 24 h, the cell viabilities were close to those of the cells incubated with phosphate-buffered saline (PBS) alone (Fig. 4c). This situation was similar for the other Fe_3_O_4_/BSA/rSiO_2_ compounds, including Fe_3_O_4_/BSA/**S**rSiO_2_, Fe_3_O_4_/BSA/**M**rSiO_2_, and Fe_3_O_4_/BSA/**C**rSiO_2_. That is, all of the cell viabilities remained at about 100% after incubation with Fe_3_O_4_/BSA/rSiO_2_ containing different length silica nanorods (Fig. 4d). It should be noted that 2 mg/mL is a high concentration for evaluating the cytotoxicity of nanoparticles. This indicated that Fe_3_O_4_/BSA before and after coating with silica rods with different lengths all showed no cytotoxicity even at high concentration. The good biocompatibility of Fe_3_O_4_/BSA/rSiO_2_ may be because Fe_3_O_4_, BSA, and SiO_2_ are all biocompatible materials.

### VMF-triggered Fe_3_O_4_/BSA/rSiO_2_ for killing cancer cells

**Fig. 5:**
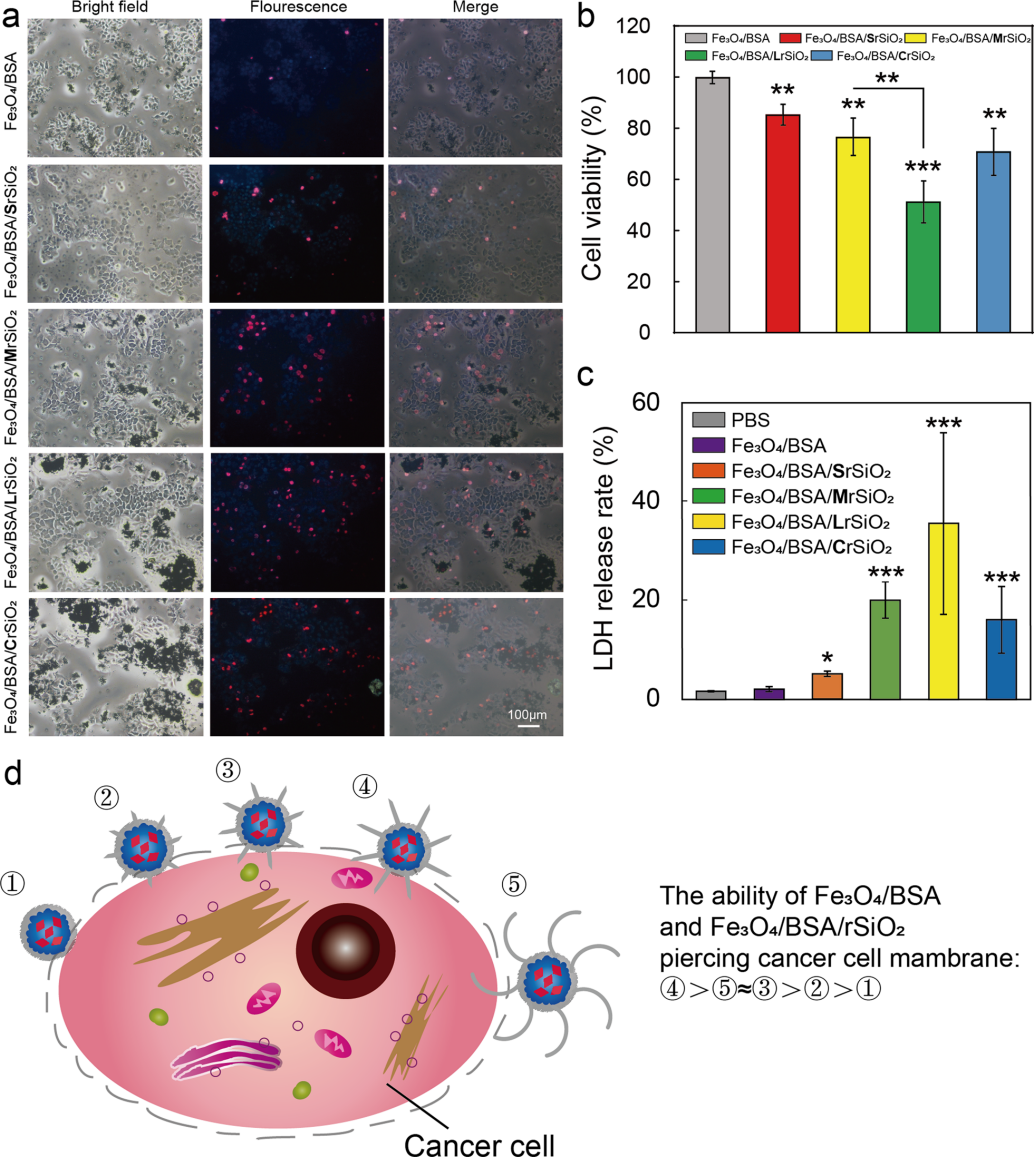
Cell killing efficiencies of Fe_3_O_4_/BSA and the four types of Fe_3_O_4_/BSA/rSiO_2_ under VMF exposure. Tu212 cells were incubated with Fe_3_O_4_/BSA and the four types of Fe_3_O_4_/BSA/rSiO_2_ (1 mg/mL for each sample) for 1 h and then exposed to a VMF (2 Hz) for 1 h. Viabilities of the Tu212 cells **a** qualitatively determined with Hoechst33342/PI and **b** quantitatively determined with CellTiter-Glo® reagent. **c** LDH leaked from the Tu212 cells. **d** Schematic illustration of the abilities of magnetic microspheres with different sharpness to pierce the cancer cell membrane. Fe_3_O_4_/BSA, Fe_3_O_4_/BSA/SrSiO_2_, Fe_3_O_4_/BSA/MrSiO_2_, Fe_3_O_4_/BSA/LrSiO_2_, and Fe_3_O_4_/BSA/CrSiO_2_ are numbered ①, ②, ③, ④, and ⑤, respectively. Significance levels observed were ***P < 0.001, **P < 0.01, and *P < 0.05

To determine whether Fe_3_O_4_/BSA/rSiO_2_ can kill cancer cells under the exposure to a VMF, Fe_3_O_4_/BSA/rSiO_2_ microspheres were added to Tu212 cells and then exposed to a VMF for 1 h. The results showed that Fe_3_O_4_/BSA/rSiO_2_ with four lengths of silica rods all efficiently induced a decrease in the cell viability (Fig. 5a and b). Furthermore, the cell killing efficiency of VMF-triggered Fe_3_O_4_/BSA/rSiO_2_ was higher than that of Fe_3_O_4_/BSA without silica rods under exposure to the same VMF. In addition, more cells were killed when the cells were incubated with Fe_3_O_4_/BSA/rSiO_2_ with longer silica rods under exposure to the same VMF. These results were confirmed by both qualitative analysis with Hoechst 33,342/propidium iodide (PI) double staining reagent (Fig. 5a) and quantitative analysis with CellTiter-Glo® reagent (Fig. 5b). This is because Fe_3_O_4_/BSA/rSiO_2_ had sharper surfaces than Fe_3_O_4_/BSA, and the microspheres with longer silica rods had more opportunity to slice more cells and/or pierce their cell membranes than those with shorter silica rods when these sharp microspheres were exposed to a VMF. For example, the viabilities of the Tu212 cells treated with Fe_3_O_4_/BSA, Fe_3_O_4_/BSA/**S**rSiO_2_, Fe_3_O_4_/BSA/**M**rSiO_2_, and Fe_3_O_4_/BSA/**L**rSiO_2_ (1 mg/mL for all samples) under the same VMF (2 Hz, 1 h) were 85.39% ± 4.12%, 76.66% ± 7.37%, 51.37% ± 8.25%, 70.94% ± 9.22%, respectively. The decrease of cell viability may be caused directly by the cell membrane damage, as PI reagent can penetrate into cells only across the damaged membranes and then “light” the cells with red fluorescence.

When the silica nanorods on the microspheres were long but curled, the cell killing efficiency of the sample (i.e., Fe_3_O_4_/BSA/**C**rSiO_2_) under VMF exposure was lower than that induced by the microspheres with long but straight silica nanorods under exposure to the same VMF (Fig. 5a and b). This may be because the sharp ends of the curled silica nanorods had fewer opportunities to pierce the cells than those of the straight silica nanorods.

When cells are damaged by a mechanical force, the cellular contents, such as lactic dehydrogenase (LDH), may be released. The LDH leakage rates induced by the VMF-triggered Fe_3_O_4_/BSA, Fe_3_O_4_/BSA/**S**rSiO_2_, Fe_3_O_4_/BSA/**M**rSiO_2_, and Fe_3_O_4_/BSA/**L**rSiO_2_ increased in order (Fig. 5c). However, the LDH leakage rate was significant lower for the Fe_3_O_4_/BSA/**C**rSiO_2_ group than that for the Fe_3_O_4_/BSA/**L**rSiO_2_ group. These results further demonstrated that the magnetic microspheres with sharp surfaces exhibited strong cell damage ability through the magneto-mechanical force.

Based on the above results, we can conclude that the order of the abilities of Fe_3_O_4_/BSA and Fe_3_O_4_/BSA/rSiO_2_ under VMF exposure for damaging cancer cells from strong to weak is Fe_3_O_4_/BSA/**L**rSiO_2_ > Fe_3_O_4_/BSA/**C**rSiO_2_ ≈ Fe_3_O_4_/BSA/**M**rSiO_2_ > Fe_3_O_4_/BSA/**S**rSiO_2_ > Fe_3_O_4_/BSA (Fig. 5d). Fe_3_O_4_/BSA/**L**rSiO_2_ was selected for subsequent cell and animal experiments.

**Fig. 6:**
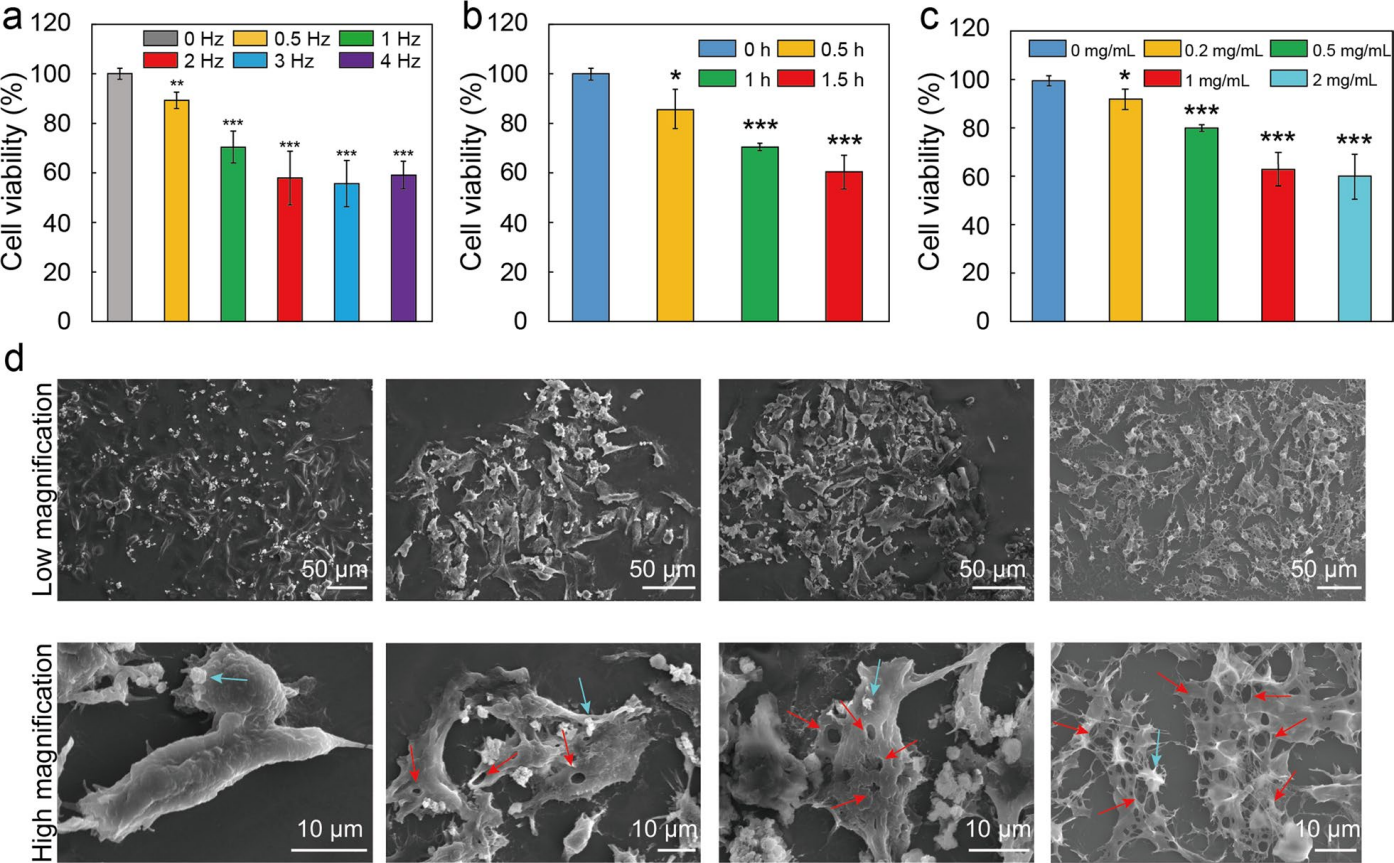
Cell killing efficiency of VMF-triggered Fe_3_O_4_/BSA/**L**rSiO_2_ under different conditions. Effects of the **a** VMF frequency, **b** VMF exposure time, and **c** Fe_3_O_4_/BSA/**L**rSiO_2_ concentration on Tu212 cell viability. In **a**, the concentration of Fe_3_O_4_/BSA/**L**rSiO_2_ and VMF exposure time were maintained at 1 mg/mL and 1 h, respectively. In **b**, the concentration of Fe_3_O_4_/BSA/**L**rSiO_2_ and VMF frequency were maintained at 1 mg/mL and 2 Hz, respectively. In **c**, the VMF frequency and exposure time were maintained at 2 Hz and 1 h, respectively. **d** SEM images of Tu212 cells incubated with Fe_3_O_4_/BSA/**L**rSiO_2_ for 1 h followed by VMF (2 Hz) exposure for 0, 0.5, 1 and 1.5 h, respectively (the red arrows indicate the representative holes caused by the mechanical force, the blue arrows indicate the representative microspheres). In all cases, the VMF strength was maintained at 400 mT. Significance levels observed were ***P < 0.001, **P < 0.01, and *P < 0.05

The cell killing efficiency improved with increasing magnetic field frequency from 0.5 to 3 Hz, but it did not further improve by continuously increasing the frequency when the cells were incubated with Fe_3_O_4_/BSA/**L**rSiO_2_ (1 mg/mL) followed by 1 h of VMF exposure (Fig. 6a). This may be because some of the magnetic microspheres with high frequency oscillation rapidly left the cells as soon as they arrived at the cell surfaces or even did not reach the cells. That is, some of the microspheres triggered by a high frequency (e.g., 4 Hz) VMF did not have the opportunity to strongly impact the cells. When the VMF exposure time was increased from 0.5 to 1.5 h (Fig. 6b) or the concentration of Fe_3_O_4_/BSA/**L**rSiO_2_ was increased from 0.2 to 1 mg/mL (Fig. 6c), the viabilities of the Tu212 cells significantly decreased. The dead cells in above groups were also confirmed by the fluorescent imaging (i.e., red fluorescent cells were dead or membrane damaged) (Additional file 1: Figs. S5–S7).

The degree of damage of the cancer cells could be clearly observed by SEM. Tu212 cells incubated with Fe_3_O_4_/BSA/**L**rSiO_2_ without VMF exposure maintained normal morphology (Fig. 6d). However, large holes appeared in the cells after the cells were exposed to a VMF, and the number and size of the holes increased with increasing VMF exposure time (Fig. 6d). For example, when the Fe_3_O_4_/BSA/**L**rSiO_2_-incubated cells were exposed to a VMF (2 Hz) for 0.5 h, many holes (most no more than ~ 2 μm in diameter) appeared in the cells. For 1.5 h VMF exposure, a large number of large holes (more than 5 μm in diameter) appeared in the cells, and some cells were nearly broken into pieces. These numerous large holes were generated by the Fe_3_O_4_/BSA/**L**rSiO_2_-mediated magneto-mechanical force, which was the direct action causing cell death.

### VMF-triggered Fe_3_O_4_/BSA/rSiO_2_ for inhibiting mouse tumor growth

**Fig. 7:**
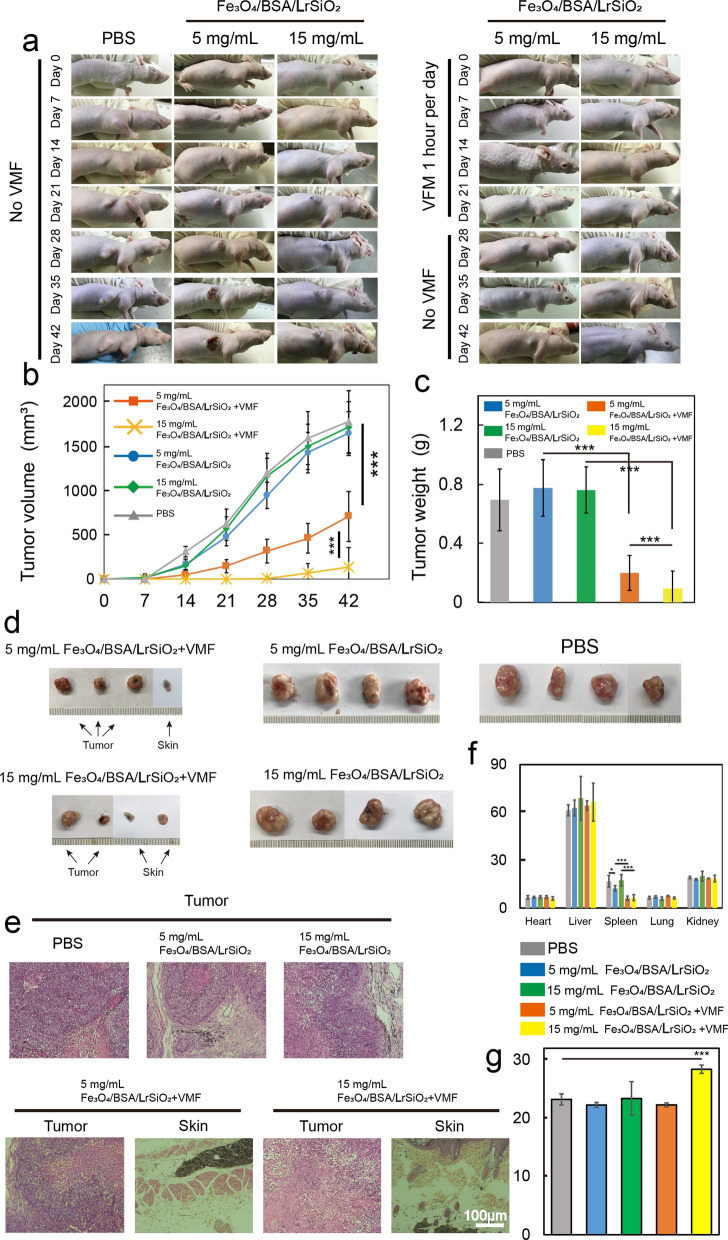
In vivo tumor inhibition by Fe_3_O_4_/BSA/**L**rSiO_2_ under VMF exposure and controls. **a** Photos of representative mice (the photos of all of the mice are shown in Additional file 1: Figs. S8–S12). **b** Tumor growth curves. **c** Tumor weights, **d** photos of the resected tumors, and **e** hematoxylin–eosin (H&E) stained images of the tumors (for the case of no tumor growth, the skin at the original injection site was resected) at 42 days post-injection. **f** Main organ coefficients and **g** mouse body weights. Significance levels observed were ***P < 0.001 and *P < 0.05

To investigate whether the VMF-triggered magnetic microspheres can inhibit tumor growth, Tu212 cells (5 × 10^6^) mixed with Fe_3_O_4_/BSA/**L**rSiO_2_ (5 mg/mL) were subcutaneously injected into nude mice. One group was exposed to a VMF for 1 h each day for 21 days (Fe_3_O_4_/BSA/**L**rSiO_2_ + VMF) and then continuously housed for 21 days. The other group was not treated with the VMF (Fe_3_O_4_/BSA/**L**rSiO_2_, no VMF). Tumor growth in the VMF group was significantly inhibited compared with the no VMF group (Fig. 7a, b and Additional file 1: Figs. S8–S12). A statistical difference in the average tumor volumes between these two groups appeared after 14 days of continuous VMF exposure. This difference in the tumor volumes continuously increased. After 21 days of VMF exposure, the average tumor volume in the VMF group was only about a third of that in the no VMF group. When the VMF was stopped from 22 to 42 days, tumor growth accelerated and the average tumor volume reached 613.991 ± 283.09 mm^3^ at the 42nd day. However, the growth rate in these days was still significant slower than that in the no VMF group (tumor volume at the 42nd day of 1650.29 ± 328.47 mm^3^). It should be noted that the cancer cells in one mouse in the VMF group were completely killed after injection of the sample, while the tumors in all of the mice in no VMF group rapidly grew after injection of the same sample.

To further investigate the ability of the Fe_3_O_4_/BSA/**L**rSiO_2_-mediated magneto-mechanical force to inhibit tumor growth, Fe_3_O_4_/BSA/**L**rSiO_2_ with higher concentration (15 mg/mL) together with Tu212 cells (5 × 10^6^) was subcutaneously injected into mice. These mice were also divided into two groups: VMF for 1 h each day for 21 days with no VMF for the subsequent 21 days and no VMF for 42 days. An interesting finding was that the tumors in all of the mice in the VMF group could almost not be detected during the first 21 days after injection, no tumors could be detected in two mice during the subsequent 21 days of no VMF exposure, and the other two mice grew only small tumors compared with the control group (Fig. 7a, b and Additional file 1: Figs. S8–S12). However, for the no VMF group, the tumors at the original injection sites rapidly grew during the 42 days after injection. In addition, Fe_3_O_4_/BSA/**L**rSiO_2_ (5 or 15 mg/mL) without VMF exposure did not inhibit tumor growth compared with the PBS group (control group).

At 42 days post-injection, the mice were sacrificed and the tumors and main organs were resected for analysis. The weights (Fig. 7c) and sizes (Fig. 7d) of the resected tumors further showed that VMF-triggered Fe_3_O_4_/BSA/**L**rSiO_2_ exhibited strong ability for inhibiting tumor growth. For example, the average tumor weight of the Fe_3_O_4_/BSA/**L**rSiO_2_ (15 mg/mL) + VMF group was only 8.13% of that of the PBS group and 22.76% of that of the Fe_3_O_4_/BSA/**L**rSiO_2_ (5 mg/mL) + VMF group. There was no statistical difference in the weights of the Fe_3_O_4_/BSA/**L**rSiO2 (no VMF) and PBS groups. The histology images showed that the tumor cells a small distance from Fe_3_O_4_/BSA/**L**rSiO_2_ in the tumor tissue were similar to those in the PBS group, which may be the reason why several tumors recovered after VMF treatment was stopped (Fig. 7e). For the groups in which no tumors could be detected, no tumor cells in the tissues at the original injection sites were observed (Fig. 7e). Generally, tumor growth can cause enlargement of the spleen owing to the immune response. Because the growth of the tumors in the two Fe_3_O_4_/BSA/**L**rSiO_2_ + VMF groups (5 and 15 mg/mL Fe_3_O_4_/BSA/**L**rSiO_2_) was significantly inhibited, the spleen coefficients (ratios of the spleen weight to the body weight) in these two groups were much smaller than those in the PBS, two Fe_3_O_4_/BSA/**L**rSiO_2_ and no VMF groups (Fig. 7f). The coefficients of the other main organs, the heart, liver, lungs, and kidneys (Fig. 7f), as well as the mouse body weights (Fig. 7g), were no statistically different among all of the groups, indicating that Fe_3_O_4_/BSA/**L**rSiO and VMF exposure did no harm to the mice.

### Magneto-mechanical force generated by Fe_3_O_4_/BSA/LrSiO_2_ exposed to a VMF

**Fig. 8:**
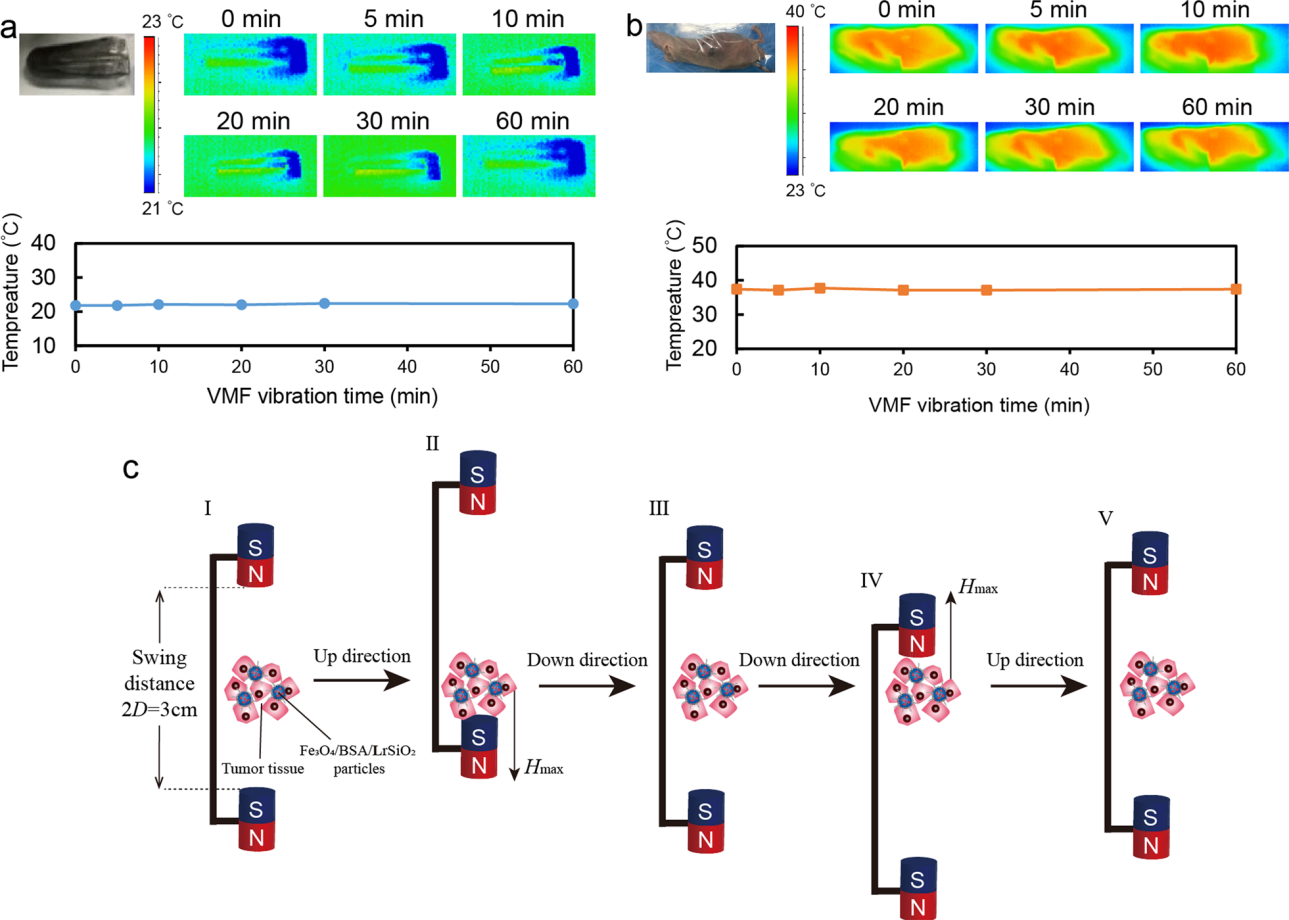
VMF-triggered Fe_3_O_4_/BSA/**L**rSiO_2_ did not cause the temperature change in **a** aqueous suspension or **b** mouse tumor. **c** Schematic illustration of the mechanical force changes generated by VMF-triggered Fe_3_O_4_/BSA/**L**rSiO_2_

No temperature increase in the Fe_3_O_4_/BSA/**L**rSiO_2_ aqueous suspension or mouse tumor containing Fe_3_O_4_/BSA/**L**rSiO_2_ was detected when the suspension or tumor was exposed to a VMF (Fig. 8a and b). The magneto-mechanical force may play an important role in in vivo tumor inhibition. The process of a Fe_3_O_4_/BSA/**L**rSiO_2_ microsphere in tumor tissue generating a force under a VMF is shown in Fig. 8c. When Fe_3_O_4_/BSA/**L**rSiO_2_ is located in the middle of the magnetic field (state I), it generates a minimal force. When the amplitude of the VMF reaches the maximum value (state II), the force also reaches the maximum value. When the VMF returns to its original location (state III) and also reaches the inverse maximum amplitude (state IV), the force reaches a minima and also reaches the inverse maximum value. After the VMF position returns to state V, the next cycle begins.

For Fe_3_O_4_/BSA/**L**rSiO_2_ microspheres in mouse tumor tissue, most of the vibrating microspheres under VMF exposure are hard to leave the cancer cell surface due to the small intercellular space. In this case, the applied magnetic force (*F*) on one microsphere is

1 $$F={\mu }_{0}\chi V{H}_{t}\nabla H$$

where *μ*_0_ = 4π × 10^−7^ (T·m·A^−1^, note: T, m and A are unites of *Tesla, metre* and *Ampere*, respectively) which is the permeability of vacuum, *V* is the volume of Fe_3_O_4_/BSA/**L**rSiO_2_ (3.35 × 10^−18^ m^3^), and *H*_t_ is the magnetic field strength on the microsphere at a certain time. $\nabla H$ is the grade of the magnetic field strength:

2 $$\nabla H = \, (H_{max} - H_{min} )/D$$

where *D* = 0.015 m (Fig. 8c), which is the distance between a magnet and one Fe_3_O_4_/BSA/**L**rSiO_2_.

In this study, the maximum and minimum magnetic field strength values (*H*_*max*_ and *H*_*min*_) were 400 mT and 0, which were at the surfaces of the two magnets and the middle position between the two magnets in the VMF equipment, respectively. Therefore, Δ*H*_*max*_ = 400 mT.

*X* is the average magnetic susceptibility of the Fe_3_O_4_/BSA/**L**rSiO_2_ microsphere under a specific magnetic field:

3 $$X \, = B/H = [{4}\pi \rho B^{\prime}/({1 } \times { 1}0^{{4}} )]/H_{max}$$

where *ρ* is the density of a Fe_3_O_4_/BSA/**L**rSiO_2_ microsphere (*ρ* = 2.72 g/cm^3^).

(Note: the unit of *B* and *H* should be the same since *X* is nondimensional. The 4π*ρB*^*’*^/(1 × 10^4^) part of (3) is to converted the unit of *B*^*’*^ from emu/g to tesla).

Based on the magnetic hysteresis loop of Fe_3_O_4_/BSA/**L**rSiO_2_ (Fig. 3), we calculated that *B*^*’*^ = 25.4 emu/g when *H* = 400 mT.

Based on the above analysis and Eq. (3), *X* = 1.1726.

The distance between a magnet and one Fe_3_O_4_/BSA/**L**rSiO_2_ microsphere at a certain time point (*D*_t_) is

4 $$D_{{\text{t}}} = D - D{\text{sin}}\omega t$$

where *ω* = 2π*f* (*f* is the magnetic field frequency), which is the rotating speed of the disk triggering the two magnets moving in straight reciprocating mode (see Scheme 1).

*H*_t_ can be considered to be the linear change between *H*_*max*_ and *H*_*min*_. Therefore, when the VMF position changes from I to III (Fig. 8c),

5 $$H_{{\text{t}}} = H_{{{\text{max}}}} \left( {D - D_{{\text{t}}} } \right)/D = H_{{{\text{max}}}} {\text{sin}}\omega t$$

When the VMF position changes from III to V (see Fig. 8c), the direction of the magnetic field changes to the opposite direction to the case from I to III. Therefore, for the whole working process of VMF,

6 $$H_{{\text{t}}} = H_{{{\text{max}}}} \mid {\text{sin}}\omega t \mid$$

Based on Eqs. (1) to (6) and the values of *μ*_0_, *X*, *V*, Δ*H*_*max*_, and *D* described above, the magneto-mechanical force is

$$F=\frac{{\mu }_{0}\chi V{H}_{max}^{2}}{D}|{\text{sin}}\omega\text{t}|= 6.17 \text{pN.}$$

This is the maximum force generated by one Fe_3_O_4_/BSA/**L**rSiO_2_ microsphere under a VMF.

In tumor tissue, the Fe_3_O_4_/BSA/**L**rSiO_2_ microspheres may always attach to the cancer cell surface or only slightly separate from the cell surface in a very short time during VMF exposure, because these microspheres are firmly trapped by surrounding cells. The magnetic field exhibits strong tissue penetration ability, and the tumor size in this study is no more than 150 mm^3^ during VMF exposure. Therefore, the force generated by Fe_3_O_4_/BSA/**L**rSiO_2_ can be completely delivered to the surrounding cancer cells.

One cancer cell at the injection site in the tumor tissue may attach more than one Fe_3_O_4_/BSA/**L**rSiO_2_ microsphere. The total force (*F*_total_) transferred from these magnetic microspheres to one cell surface is

7 $$F_{\text{total}} = 6.17 \times n{\text{pN}}$$

where *n* is the number of Fe_3_O_4_/BSA/**L**rSiO_2_ microspheres on the cell surface. Therefore, the Fe_3_O_4_/BSA/**L**rSiO_2_-mediated force acting on one cell may be tens or more than one hundred of piconewtons. The cancer cells will be induced to death by such a large force, as it has been demonstrated that tens of piconewtons [1] or even smaller force [18] generated by one particle could damage cells.

### Intracellular ROS generated by Fe_3_O_4_/BSA/LrSiO_2_ exposed to a VMF

**Fig. 9:**
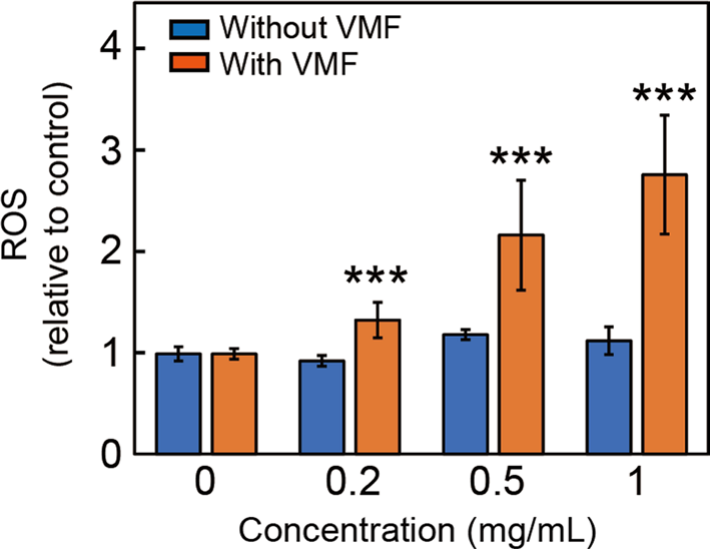
Intracellular ROS levels generated by VMF-triggered Fe_3_O_4_/BSA/**L**rSiO_2_ and controls. Significance levels observed was ***P < 0.001

It was an interesting result that Fe_3_O_4_/BSA/rSiO_2_ exposed to a VMF generated intracellular ROS. When the concentration of Fe_3_O_4_/BSA/**L**rSiO_2_ in the cells was 0.2, 0.5, and 1 mg/mL and the VMF (400 mT, 2 Hz) exposure time was 1 h, the ROS levels were all higher than that in the control group (cells only), and the ROS level increased with increasing Fe_3_O_4_/BSA/**L**rSiO_2_ concentration (Fig. 9). For example, when 1 mg/mL of Fe_3_O_4_/BSA/**L**rSiO_2_ was added to Tu212 cells and triggered with the VMF for 1 h, the ROS level was ~ 2.08 times higher than that of 0.2 mg/mL Fe_3_O_4_/BSA/**L**rSiO_2_ under the same VMF and ~ 3.08 times higher than that of the control. For the cases where the cells were incubated with different Fe_3_O_4_/BSA/**L**rSiO_2_ concentrations without VMF exposure and the cells alone were exposed to the VMF for 1 h, nearly no ROS were detected compared with the control.

The magneto-mechanical force induced ROS generation, which may be because the cells were mechanically damaged, resulted in an imbalance between production and elimination of oxygen free radicals in the cells. The abnormal cell metabolism may result in ROS such as hydrogen peroxide accumulating in cells. The damaged cellular microenvironment infiltrated the cells, and Fe_3_O_4_ in Fe_3_O_4_/BSA/rSiO_2_ catalyzed hydrogen peroxide to further generate ROS (hydroxyl radicals). ROS exhibit high toxicity to cells and tissues through oxidative damage of the proteins and lipids in the cells, such as cutting off the peptide chains in proteins. Therefore, the VMF-triggered Fe_3_O_4_/BSA/**L**rSiO_2_ killing of cancer cells and inhibition of tumor growth described above may be because of the combination of the magneto-mechanical force and force-induced ROS (e.g., FDT).

### Toxicity of Fe_3_O_4_/BSA/LrSiO_2_ to mice

**Fig. 10:**
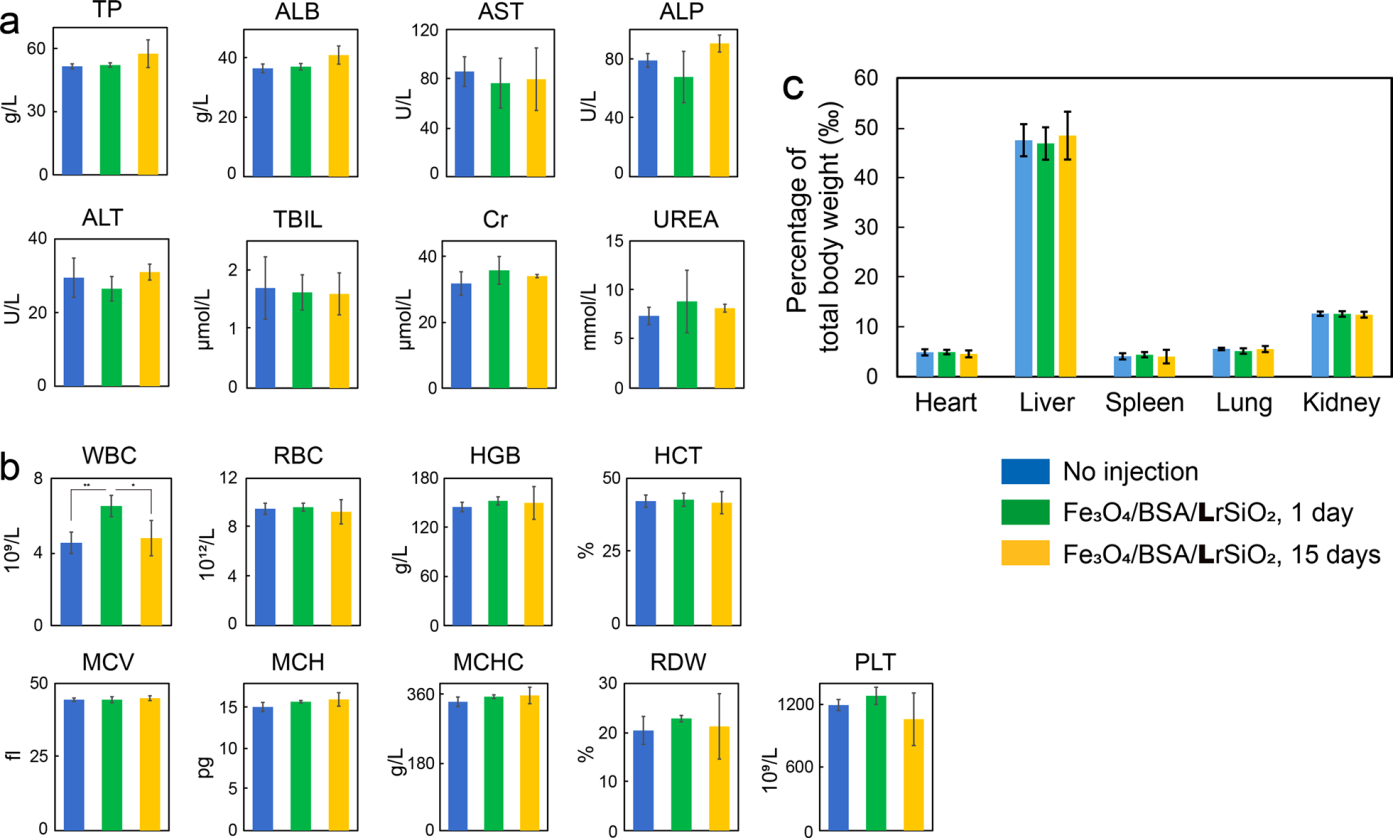
Blood biochemistry, hematology levels, and main organ coefficients of the mice at different times post-injection of Fe_3_O_4_/BSA/LrSiO_2_ and the controls. **a** Levels of the serum biochemistry parameters, including albumin (ALB), globulin (GLOB), albumin/globulin (A/G) ratio, aspartate aminotransferase (AST), alanine aminotransferase (ALT), alkaline phosphatase (ALP), total protein (TP), creatinine (CRE), and urea. **b** Levels of the blood routine parameters, including WBCs, red blood cells (RBCs), hematocrit (HCT), mean corpuscular volume (MCV), mean corpuscular hemoglobin (MCH), mean corpuscular hemoglobin concentration (MCHC), red cell distribution width (RDW), hemoglobin (HGB), and platelets (PLTs). **c** Main organ coefficients (weight ratios of the organ weights to the total body weight). Significance levels observed were **P < 0.01, and *P < 0.05

The eight parameter levels of the liver and kidney functions of mice injected with Fe_3_O_4_/BSA/**L**rSiO_2_ at both 1 day and 15 days post-injection were all in the normal ranges, and they were not statistically significant different from those of mice injected with PBS (control) (Fig. 10a). At 1 day post-injection, the average white blood cell (WBC) count was significantly higher than that of the control (Fig. 10b). This may be because the local damage caused by injection of Fe_3_O_4_/BSA/**L**rSiO_2_ induced an immune response, and more WBCs were generated for wound repair. At 15 days post-injection, the WBC count returned to normal. The other eight blood routine parameters at both 1 day and 15 days post-injection were all not statistically different from those of the control group, and they were all located in the normal ranges (Fig. 10b).

The sizes and weights of the mouse spleens in the Fe_3_O_4_/BSA/**L**rSiO_2_ group 15 days post-injection were similar with those in the PBS group (Fig. 10c), which indicated that there was no significant inflammatory response at this time. This result further demonstrated that the WBC increase mentioned above was a temporary phenomenon in the early stage after injection. There were no statistically significant differences in the main organ (including spleen) coefficients between the Fe_3_O_4_/BSA/**L**rSiO_2_ and PBS groups (Fig. 10c). These results indicated that the mice injected with Fe_3_O_4_/BSA/**L**rSiO_2_ were in good health.

## Conclusions

Superparamagnetic Fe_3_O_4_/BSA/rSiO_2_ hybridized microspheres with a flagellum-like surface have been synthesized. The obtained magnetic microspheres could be used for killing cancer cells in vitro and inhibiting mouse tumor growth through the magneto-mechanical force and FDT. The magnetic microspheres coated with long straight silica rods (i.e., Fe_3_O_4_/BSA/**L**rSiO_2_) exposed to a VMF of only several hertz exhibited the strongest ability for cell killing cancer cells among the microspheres. The cell killing efficiency increased with increasing magnetic field frequency (≤ 3 Hz), VMF exposure time (≤ 1.5 h) and microsphere concentration (≤ 1 mg/mL). VMF-triggered Fe_3_O_4_/BSA/**L**rSiO_2_ remarkably interfered with mouse tumor growth, and no tumor could be detected if the dose of Fe_3_O_4_/BSA/**L**rSiO_2_ attained an appropriate concentration. Because Fe_3_O_4_/BSA/rSiO_2_ microspheres show good biocompatibility and the magneto-mechanical force-mediated cell killing method is not selective for cancer cell types or affected by the tumor microenvironment, the reported strategy may be suitable for treating all types of solid tumors.

## Supplementary Information

**Additional file 1: Figure S1.** Additional experimental methods and figures.

**Additional file 2. **Fe3O4-BSA-rSiO2 microspheres vibrating under a VMF.

## Data Availability

The data used to support the findings of this study are available from the corresponding author upon request.

## Abbreviations

BSA: Bovine serum albumin
VMF: Vibrating magnetic field
ROS: Reactive oxygen species
FDT: Force-dynamic therapy
TEOS: Tetraethyl orthosilicate
FeSO4·7H2O: Ferrous sulfate
NaOH: Sodium hydroxide
PVP: Polyvinyl pyrrolidone
PI: Propidium iodide
DCFH-DA: 2,7-Dichlorofluorescin diacetate
H&E: Hematoxylin and eosin
Tu212: Human laryngeal carcinoma cells
FBS: Fetal bovine serum
PBS: Phosphate-buffered saline
HRTEM: High-resolution transmission electron microscopy
EDS: Energy-dispersive spectroscopy
SEM: Scanning electron microscopy
EDX: X-ray spectroscopy
LDH: Lactic dehydrogenase
ALB: Albumin
GLOB: Globulin
A/G: Albumin/globulin
AST: Aspartate aminotransferase
ALT: Alanine aminotransferase
ALP: Alkaline phosphatase
TP: Total protein
CRE: Creatinine
RBCs: Red blood cells
HCT: Hematocrit
MCV: Mean corpuscular volume
MCH: Mean corpuscular hemoglobin
MCHC: Mean corpuscular hemoglobin concentration
RDW: Red cell distribution width
HGB: Hemoglobin
PLTs: Platelets
