# Correction: Low frequency vibrating magnetic field-triggered magnetic microspheres with a nanoflagellum-like surface for cancer therapy

**DOI:** 10.1186/s12951-026-04713-7

**Published:** 2026-07-15

**Authors:** Yuliang Guo, Wenxuan Yang, Guangjin Pu, Chunjiao Zhu, Yifan Zhu, Ji Li, Yuqiao Huang, Bo Wang, Maoquan Chu

**Affiliations:** 1 Research Center for Translational Medicine at Shanghai East Hospital, School of Life Sciences and Technology, Tongji University, Shanghai, 200092 People’s Republic of China; 2 School of Physics Science and Engineering, Tongji University, Shanghai, 200092 People’s Republic of China

**Correction: Journal of Nanobiotechnology (2022) 20:316**

**https://doi.org/10.1186/s12951-022-01521-7**

In this article Figs. 5 and 7 appeared incorrectly. Two images in this article were incorrectly selected from the original data. These images should be replaced by the corrected images, as detailed below: In Fig. 5a, the cell images representing the Fe3O4/BSA/SrSiO2 group were incorrectly selected from the original sample source. In Fig. 7e, the H&E staining image of tumor representing the “15 mg/mL Fe3O4/BSA/SrSiO2 + VMF” group was incorrectly selected from the original sample sourceFor completeness and transparency, the incorrect and correct versions are displayed below.

Incorrect Fig. 5:

**Figure:**
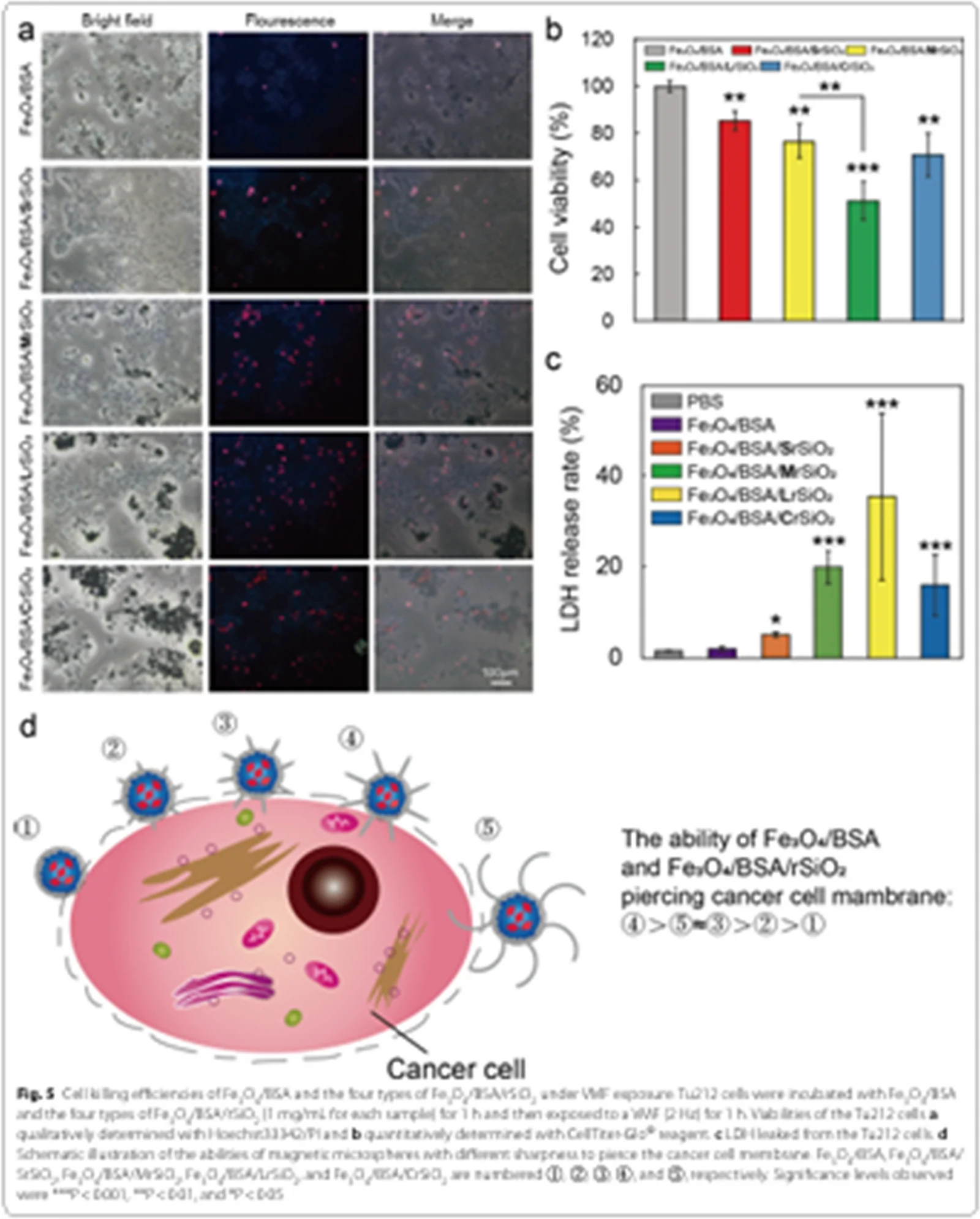

Correct Fig. 5:

**Fig. 5:**
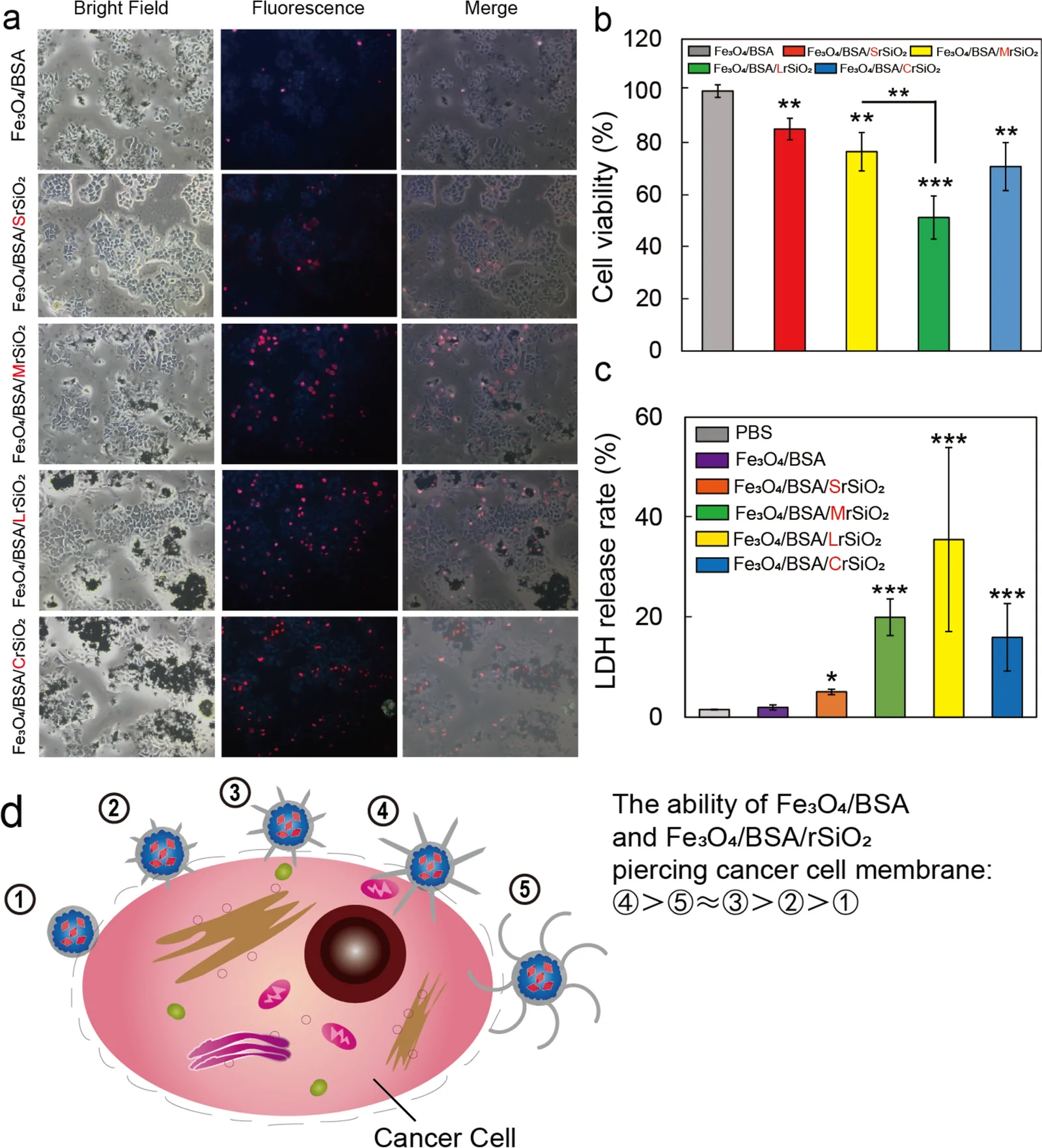
Cell killing efficiencies of Fe3O4/BSA and the four types of Fe3O4/BSA/rSiO2 under VMF exposure. Tu212 cells were incubated with Fe3O4/BSA and the four types of Fe3O4/BSA/rSiO2 (1 mg/mL for each sample) for 1 h and then exposed to a VMF (2 Hz) for 1 h. Viabilities of the Tu212 cells **a** qualitatively determined with Hoechst33342/PI and **b** quantitatively determined with CellTiter-Glo® reagent. **c** LDH leaked from the Tu212 cells. **d** Schematic illustration of the abilities of magnetic microspheres with different sharpness to pierce the cancer cell membrane. Fe3O4/BSA, Fe3O4/BSA/SrSiO2, Fe3O4/BSA/MrSiO2, Fe3O4/BSA/LrSiO2, and Fe3O4/BSA/CrSiO2 are numbered ①, ②, ③, ④, and ⑤, respectively. Significance levels observed were ***P < 0.001, **P < 0.01, and *P < 0.05

Incorrect Fig. 7:

**Figure:**
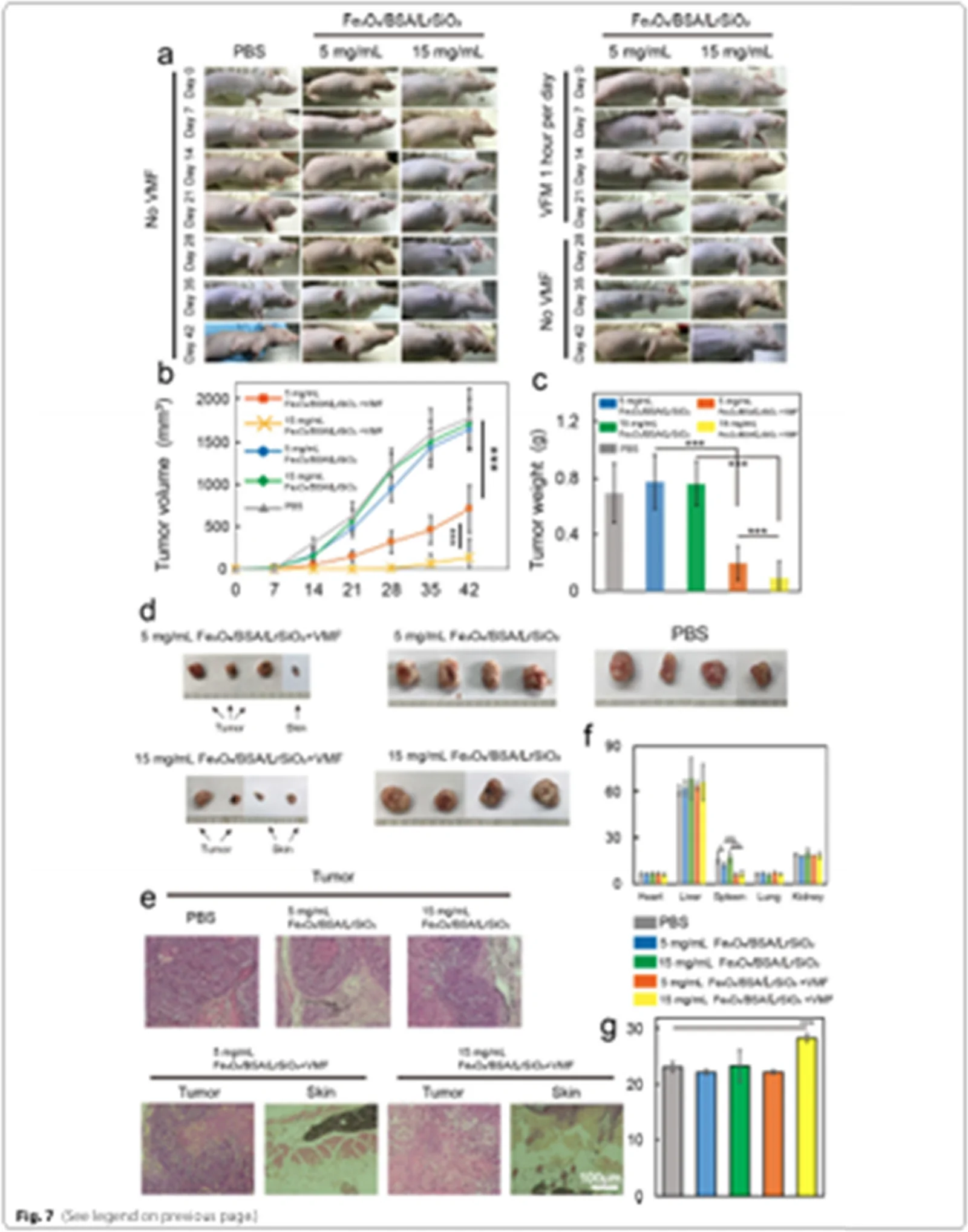

Correct Fig. 7:

**Fig. 7:**
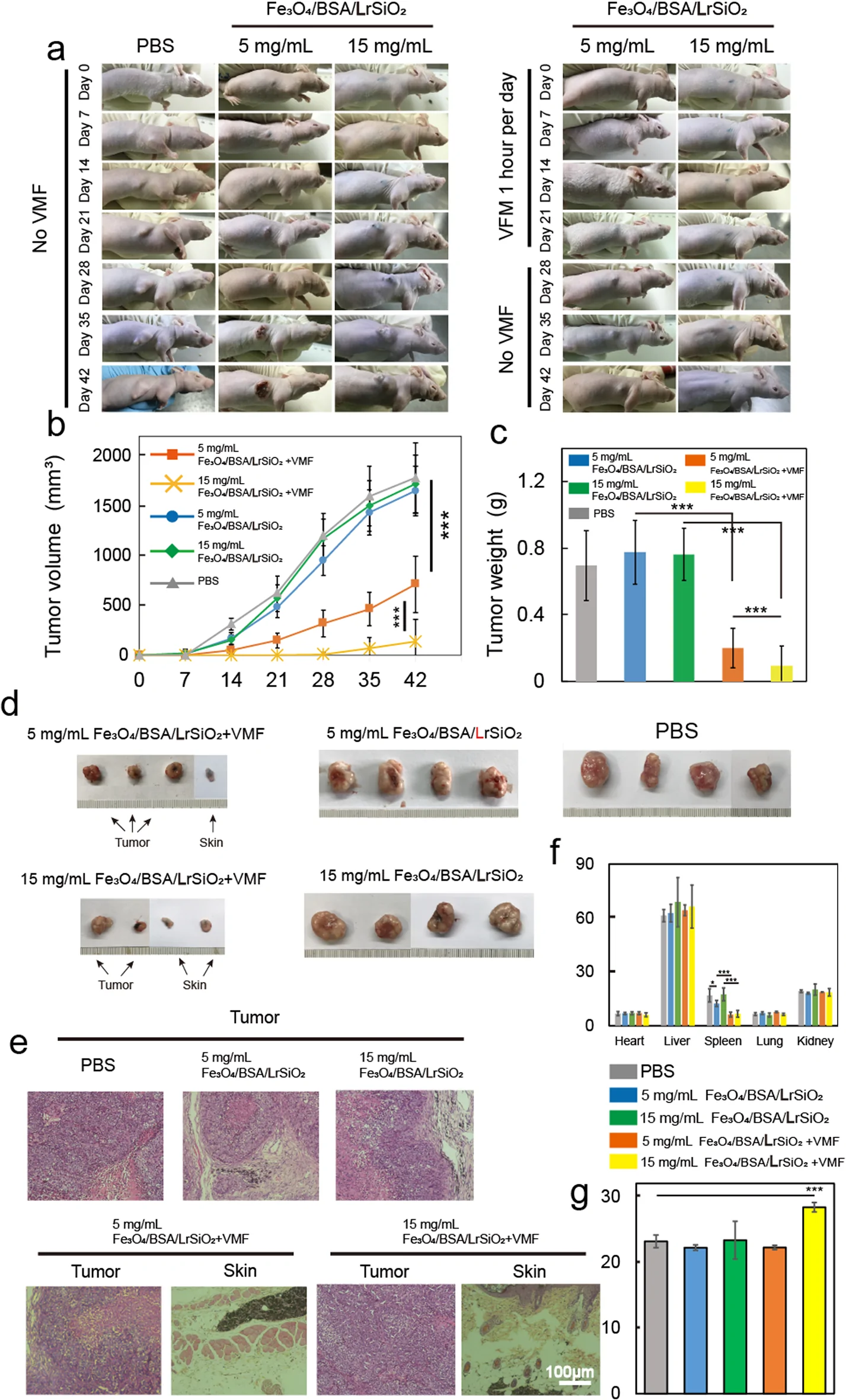
In vivo tumor inhibition by Fe3O4/BSA/LrSiO2 under VMF exposure and controls. **a** Photos of representative mice (the photos of all of the mice are shown in Additional file 1: Figs. S8–S12). **b** Tumor growth curves. **c** Tumor weights, **d** photos of the resected tumors, and **e** hematoxylineosin (H&E) stained images of the tumors (for the case of no tumor growth, the skin at the original injection site was resected) at 42 days post-injection. **f** Main organ coefficients and **g** mouse body weights. Significance levels observed were ***P < 0.001 and *P < 0.05

The original article has been corrected.

