# Supplementary material for: Low frequency vibrating magnetic field-triggered magnetic microspheres with a nanoflagellum-like surface for cancer therapy
Source: J Nanobiotechnology. 2022 Jul 6;20:316. doi: 10.1186/s12951-022-01521-7 (PMC9258173; doi:10.1186/s12951-022-01521-7)
Supplement: Supplementary file 1 — Additional file 1: Figure S1. Additional experimental methods and figures. [file 12951_2022_1521_MOESM1_ESM.docx]

**Supplementary Material**

Low Frequency Vibrating Magnetic Field-Triggered Magnetic Microspheres with a Nanoflagellum-like Surface for Cancer Therapy

*Yuliang Guo^1^, Wenxuan Yang^1^, Guangjin Pu^1^, Chunjiao Zhu^1^, Yifan Zhu^2^, Ji Li^1^, Yuqiao Huang^1^, Bo Wang^1^, Maoquan Chu**^1,^**

1. Research Center for Translational Medicine at Shanghai East Hospital, School of Life Sciences and Technology, Tongji University, Shanghai 200092, P. R. China.

2. School of Physics Science and Engineering, Tongji University, Shanghai 200092, P. R. China.

* Corresponding author: Tel: 86 21 65988653. Email: mqchu98@tongji.edu.cn

**1. Supplementary Materials**

1.1. Materials

BSA (purity: >99%) was purchased from Bovogen Biologicals Pty Ltd. (Ogilvie St, Essendon, Australia). Tetraethyl orthosilicate (TEOS) (purity: GR) and ammonia were purchased from TCI Development Co., Ltd. (Shanghai, China). Ferrous sulfate (FeSO_4_·7H_2_O)、sodium hydroxide (NaOH)、ethanol absolute、1-Pentanol、trisodium citrate dihydrate and polyvinyl pyrrolidone (PVP) and Hoechst 33342/propidium iodide (PI) were bought from Sinopharm Chemical Reagent Co., Ltd (Shanghai, China). CellTiter-Glo® luminescent cell viability assay reagents were purchased from Promega Corporation (Madison, WI, USA). RPMI-1640 culture medium and fetal calf serum were obtained from Hyclone (Logan, UT, USA). Lactate dehydrogenase (LDH) and 2,7-dichlorofluorescin diacetate (DCFH-DA) were acquired from Beyotime Institute of Biotechnology (Jiangsu, China). The hematoxylin and eosin (H&E) staining kit was purchased from Shanghai Biyuntian Biological Co., Ltd. (Shanghai, China). Other reagents, if not mentioned separately, were bought from Sinopharm Chemical Reagent Co., Ltd (Shanghai, China). All reagents were used directly as received without any further purification.

1.2. Cell line and animals

Human laryngeal carcinoma cells (Tu212) were purchased from Shanghai EK-Bioscience Biotechnology Co., Ltd. (Shanghai, China). Nude mice and ICR mice (aged 5-6 weeks, male) were purchased from Shanghai SIPPR-BK Laboratory Animal Co., Ltd. (Shanghai, China). Mice were housed and fed in a specific pathogen-free environment, with plenty supplement of food and drinking water. All animal experiments were performed in accordance with the University of Tongji Institutional Animal Care and Use Committee Guidelines (No: TJAB05820102).

**2. Supplementary Experimental Methods**

**2.1. Characterizations of Fe_3_O_4_, Fe_3_O_4_/BSA and Fe_3_O_4_/BSA/rSiO_2_**

The morphologies of samples including Fe_3_O_4_ nanoparticles, Fe_3_O_4_/BSA and Fe_3_O_4_/BSA/rSiO_2_ microspheres were observed using a transmission electron microscopy (TEM; JEOL-1230, Tokyo, Japan) and field-emission scanning electron microscope (SEM; S-4800; Hitachi, Japan) (except Fe_3_O_4_ nanoparticles). Size distributions of silica rod length on Fe_3_O_4_/BSA surface were detected from TEM images of over 30 rods for each sample. X-ray diffraction (XRD) of Fe_3_O_4_ nanoparticles were detected on a Rigaku D/max 2550 VB/PC X-ray diffractometer (Tokyo, Japan) using Cu K_α_ radiation. Energy-dispersive X-ray spectroscopy (EDS) and elemental mapping of Fe_3_O_4_/BSA/rSiO_2_ were detected using a high-resolution transmission electron microscope (HRTEM; JEM-1230; JEOL, Japan). Magnetisms of Fe_3_O_4_, Fe_3_O_4_/BSA and four kinds of Fe_3_O_4_/BSA/rSiO_2_ were detected using a vibrating sample magnetometer (Physical Property Measurement System, Quantum Design, USA).

**2.2. Detection of the cytotoxicity of Fe_3_O_4_/BSA/rSiO_2_**

*2.2.1. Cell cycle distribution*

The effect of Fe_3_O_4_/BSA/rSiO_2_ on the cell cycle distribution was determined as follows. Tu212 cells (~1 × 10^4^) were cultured in RPMI-1640 medium in a 96-well plate. After culturing in a CO_2_ incubator at 37 °C for about 24 h, the medium was removed and the cells were washed three times with PBS. The medium was removed and RPMI-1640 medium-dispersed Fe_3_O_4_/BSA/rSiO_2_ with long silica rods (2 mg/mL, 100 μL) was added to the cells followed by culturing in a CO_2_ incubator for 48 h. The cells were then digested and fixed with 70% ethanol for 2 h, followed by detection using a Cell Cycle Analysis Kit (Beyotime Biotechnology, Shanghai, China). The four major phases of the cell cycle distribution, the G1, S, G2, and M phases, were analyzed by the fluorescence detected by analytical flow cytometry (BD FACSVerse, Becton, Dickinson and Company, Franklin Lakes, USA). The cells without addition of the samples were used as the control. Each experiment was repeated three times.

*2.2.2. Detection of the effect of Fe_3_O_4_/BSA/rSiO_2_ on cell viability*

The effect of Fe_3_O_4_/BSA/rSiO_2_ on cell cycle distribution was detected as follows. Tu212 cells (~1 × 10^4^) were cultured in RPMI-1640 medium in a 96-well plate in a CO_2_ incubator at 37 °C. After culture for 24 h, the medium was removed and serum-free medium-dispersed Fe_3_O_4_/BSA and Fe_3_O_4_/BSA/rSiO_2_ with short, medium, long and curling silica rods (2 mg/mL for each samples, 100 uL for each well) were added to the cells and cultured in the CO_2_ incubator for 2 or 24 h. Each sample for each culture period was distributed to five wells. The cells in five wells in each 96-well plate without addition of samples were as controls. The cell viabilities were detected using CellTiter-Glo® reagent.

**2.3. Measurement of LDH leaked from cancer cells damaged by the Fe_3_O_4_/BSA/rSiO_2_ triggered by a VMF**

The Tu212 cells were cultured in a 96-well plate using the same manner as described above. Serum-free medium-dispersed Fe_3_O_4_/BSA and Fe_3_O_4_/BSA/rSiO_2_ with different lengths of silica rods (1 mg/mL for each samples, 100 μL for each well) were incubated with cells at 37 °C for 1 h, followed by 1 h of VMF (2 Hz) exposure. The cells without the treatment of Fe_3_O_4_/BSA/rSiO_2_ or VMF exposure were as controls. Four hours after VMF exposure, the LDH leakage was measured by detection of the absorption intensities (AI) at 490 nm of cell culture media using a LDH cytotoxicity assay kit. The leakage rate (LR) was calculated by the following equation: LR= [(AI_(treated cells)_- AI_(control cells)_)/(AI_(maximal activity cells)_- AI_(control cells)_)] × 100%. Each experiment was repeated five times.

**2.4. Study the effect of Fe_3_O_4_/BSA/LrSiO_2_ on mouse kidney and liver functions and hematology parameters**

PBS-dispersed Fe_3_O_4_/BSA/**L**rSiO_2_ microspheres (15 mg/mL, 50 μL for each mouse) were subcutaneously injected into eight ICR mice. The mice were sacrificed at 1 and 15 days post-injection (*n*=4 for each time point), and the blood was extracted from the retro-orbital sinus of mice. Each blood sample was divided into two aliquots. One was used to detect the following eight chemistry parameters including albumin (ALB), globulin (GLOB), albumin/globulin ratio (A/G), aspartate aminotransferase (AST), alanine aminotransferase (ALT), alkaline phosphatase (ALP), total protein (TP), creatinine (CRE) and urea. Another was used to detect the following nine hematology parameters including white blood cells (WBC), red blood cells (RBC), hematocrit (HCT), mean corpuscular volume (MCV), mean corpuscular hemoglobin (MCH), mean corpuscular hemoglobin concentration (MCHC),  red cell distribution width (RDW), hemoglobin (HGB) and platelets (PLT). The bloods extracted from the mice (*n*=4) injected with PBS were as controls. All these parameters were measured at KingMed Diagnostics (Shanghai, China).

2.5. Statistical analysis

Significant differences between groups were determined using the one-way ANOVA test in Excel 2013 (Microsoft, Redmond, USA). A P-value < 0.05 was considered statistically significant, P < 0.01 was considered highly significant, P < 0.001 was considered very highly significant. Data are expressed as mean ± standard deviation.

**3. Supplementary Figures**


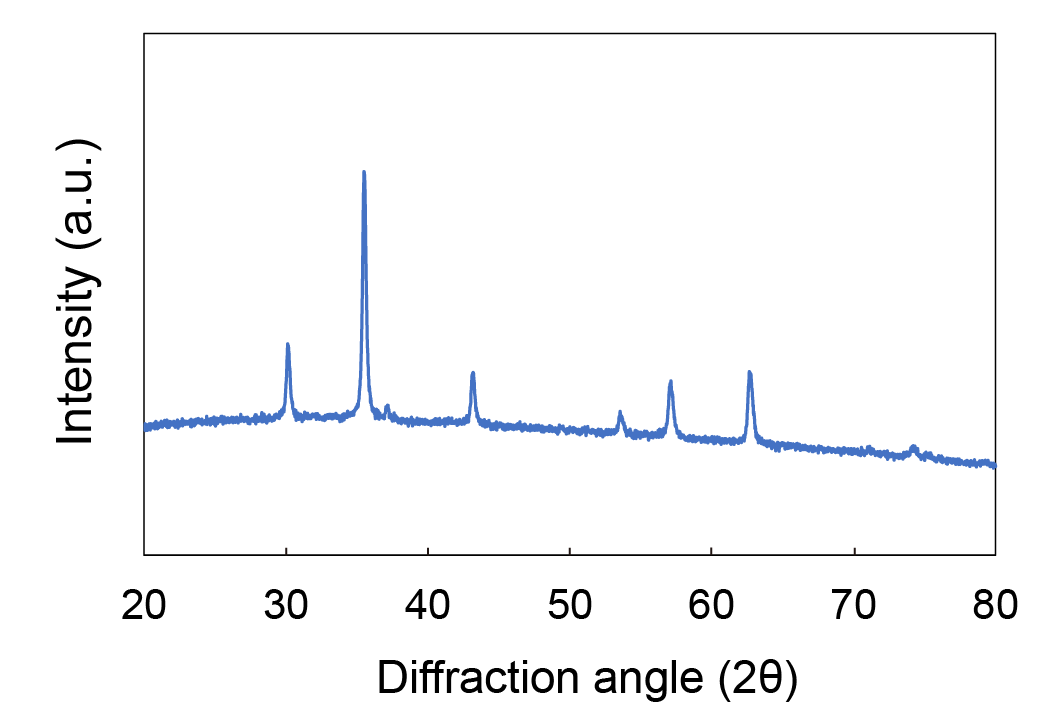


**Figure S1.** XRD pattern of Fe_3_O_4_ nanoparticles


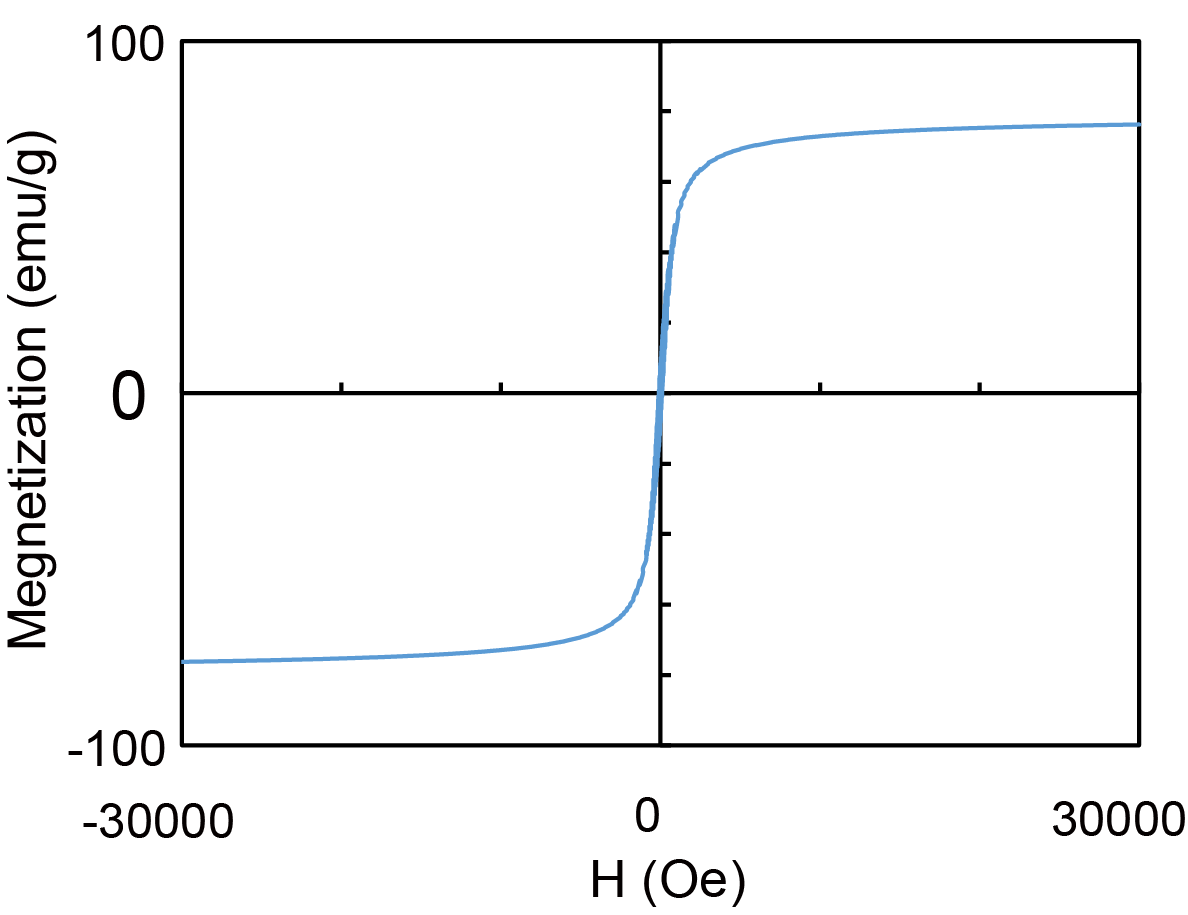


**Figure S2.** Magnetization hysteresis of Fe_3_O_4_


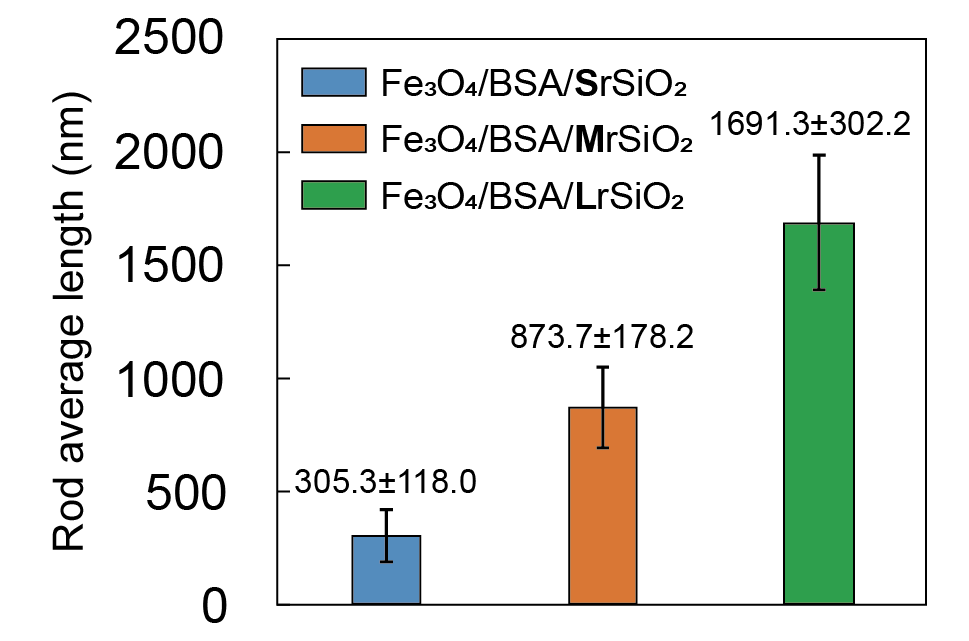


**Figure S3.** Average silica nanorod lengths of Fe_3_O_4_/BSA/**S**rSiO_2_, Fe_3_O_4_/BSA/**M**rSiO_2_, Fe_3_O_4_/BSA/**L**rSiO_2_.


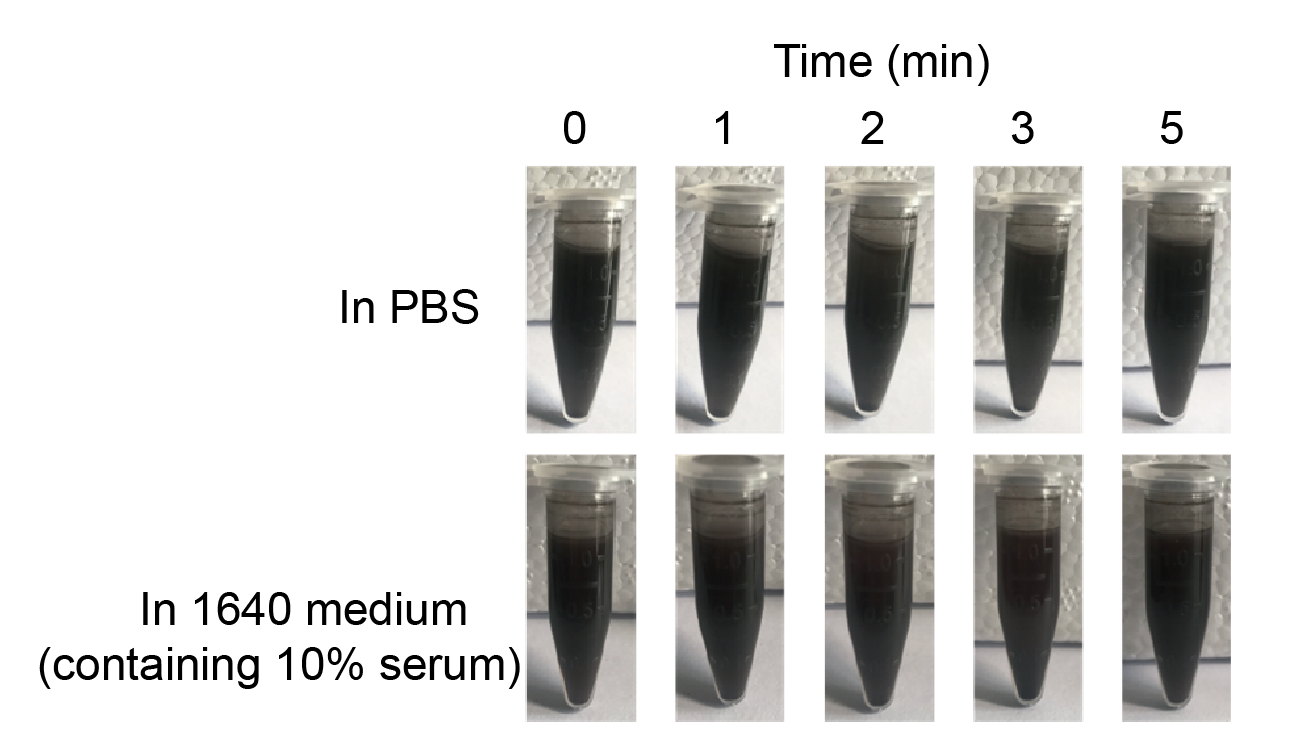


Figure S4. Photographs of the stability of Fe_3_O_4_/BSA/**L**rSiO_2_ dispersed in PBS or cell culture medium


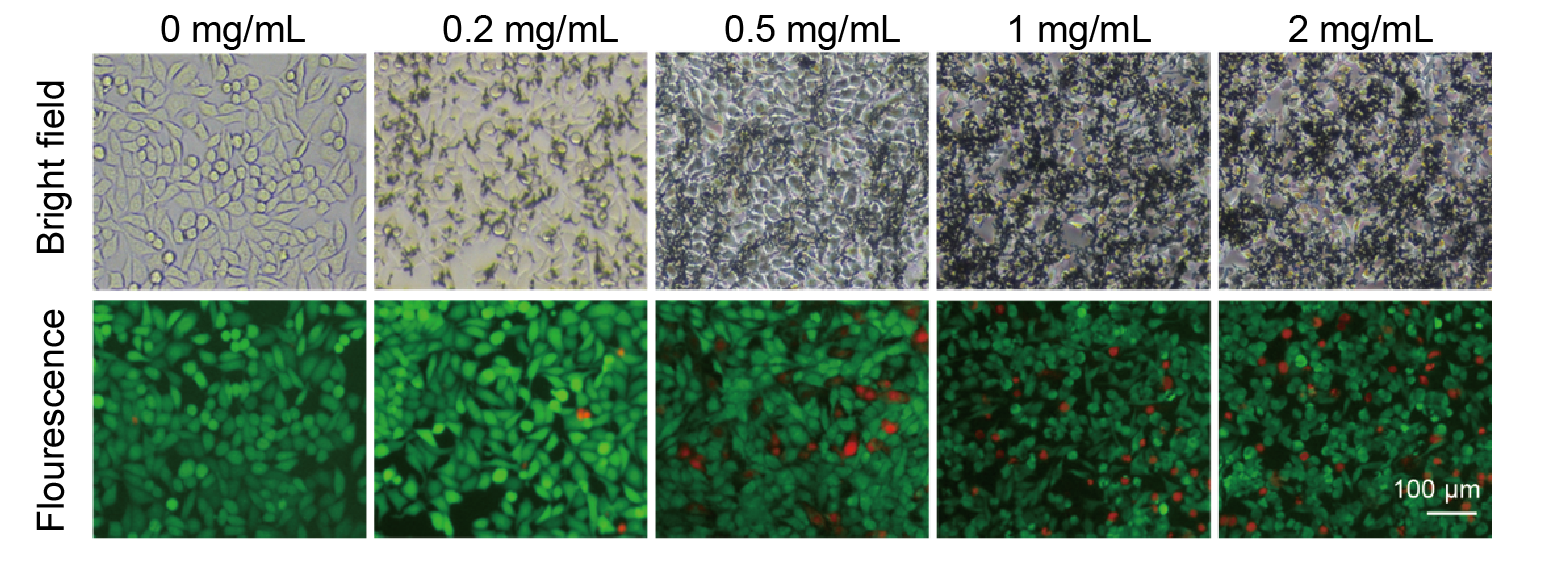


Figure S5. TU212 cells were damaged by the VMF-triggered Fe_3_O_4_/BSA/**L**rSiO_2_ with different concentrations (VMF: 2 Hz, 1 h). The damaged cells were detected using Calcein-AM/PI double-staining method.


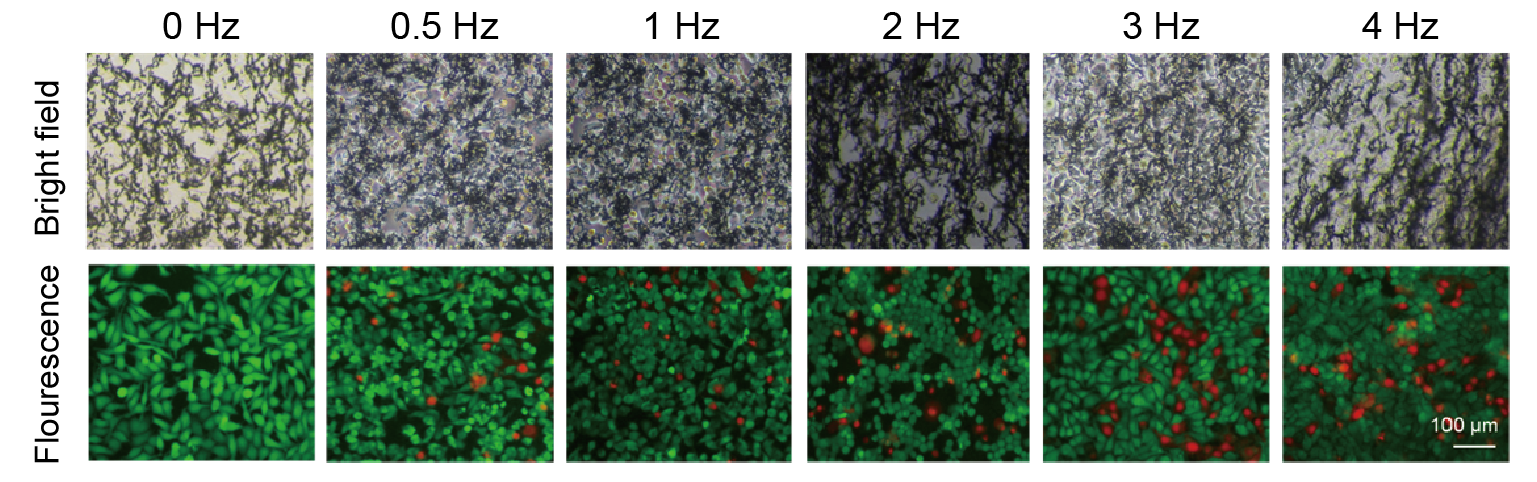


Figure S6. TU212 cells were damaged by the VMF-triggered Fe_3_O_4_/BSA/**L**rSiO_2_ under the condition of different VMF frequencies (Fe_3_O_4_/BSA/**L**rSiO_2_: 1 mg/mL, VMF: 1 h). The damaged cells were detected using Calcein-AM/PI double-staining method.


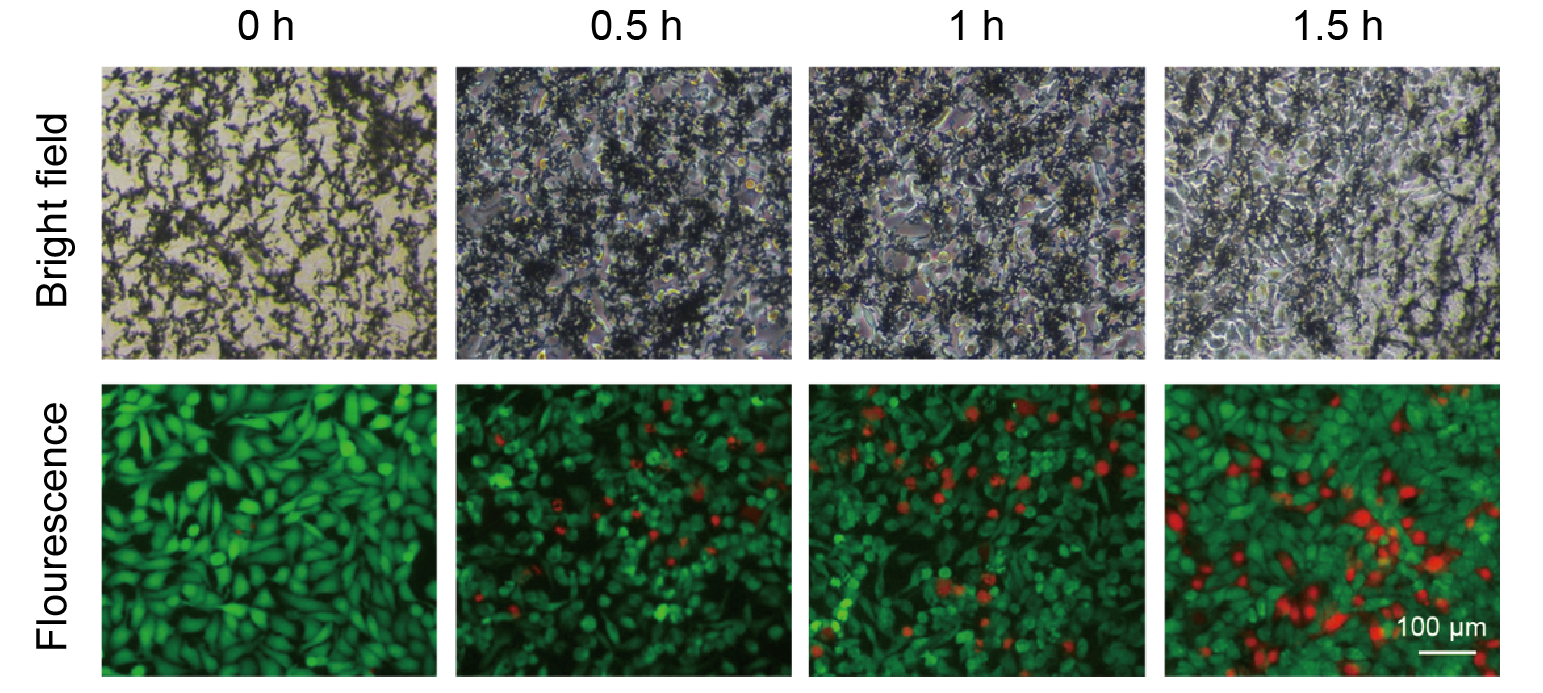


Figure S7. TU212 cells were damaged by the Fe_3_O_4_/BSA/**L**rSiO_2_ under VMF exposure for different time (Fe_3_O_4_/BSA/**L**rSiO_2_: 1 mg/mL, VMF: 2 Hz). The damaged cells were detected using Calcein-AM/PI double-staining method.


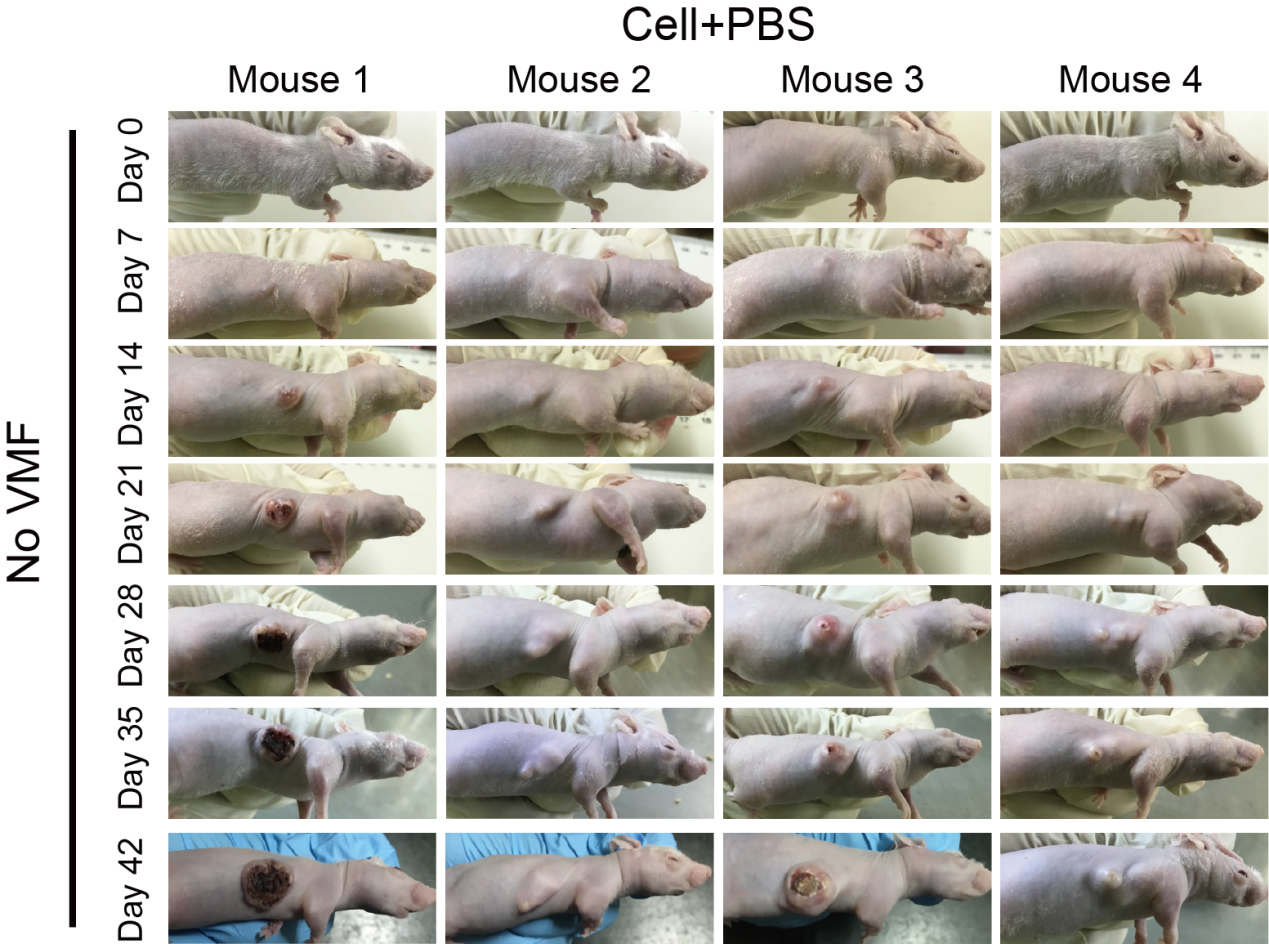


**Figure S8.** Tumor-bearing mice without treatment


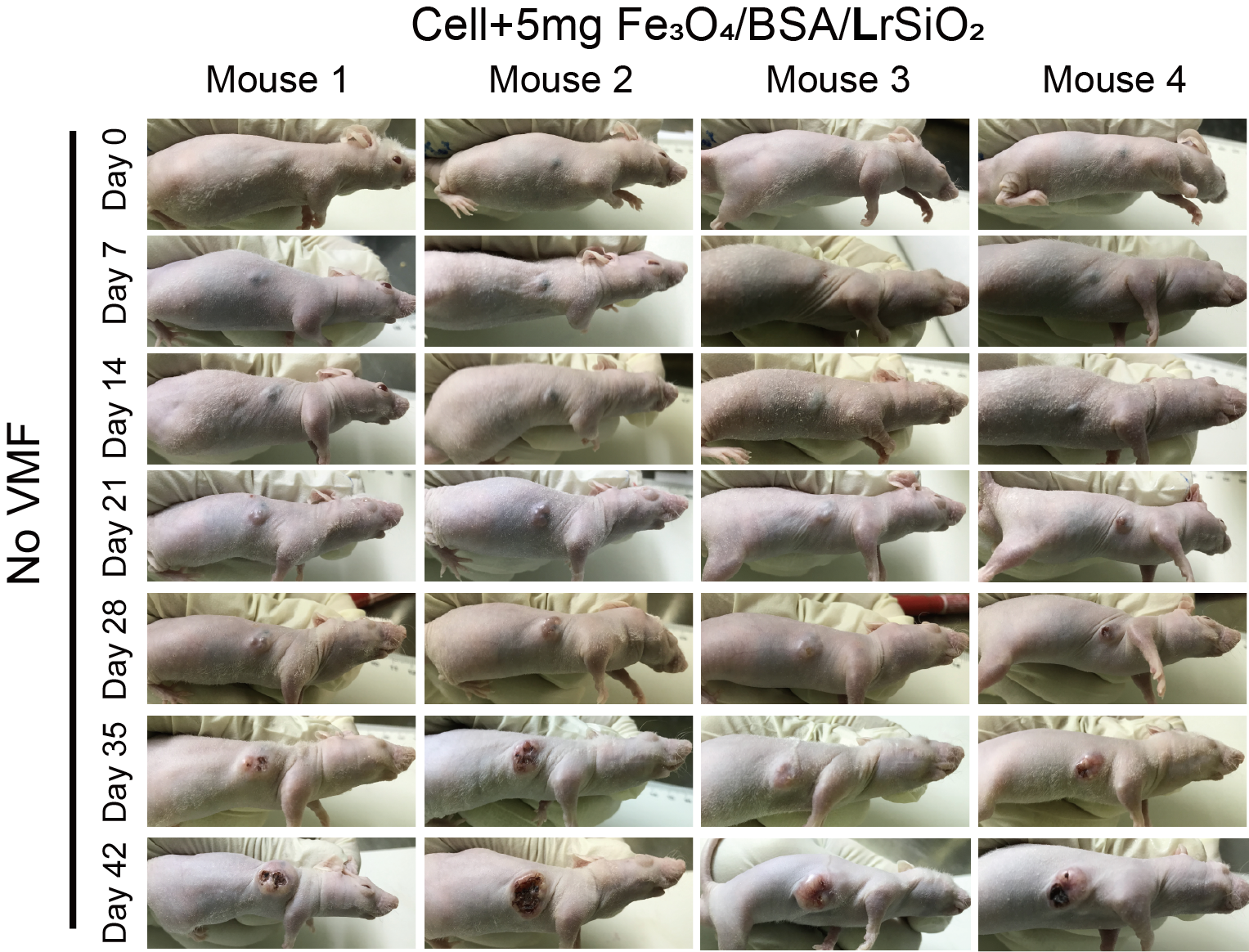


**Figure S9.** Tumor-bearing mice treated with 5 mg/mL Fe_3_O_4_/BSA/**L**rSiO_2_ (50 μL for each mouse)


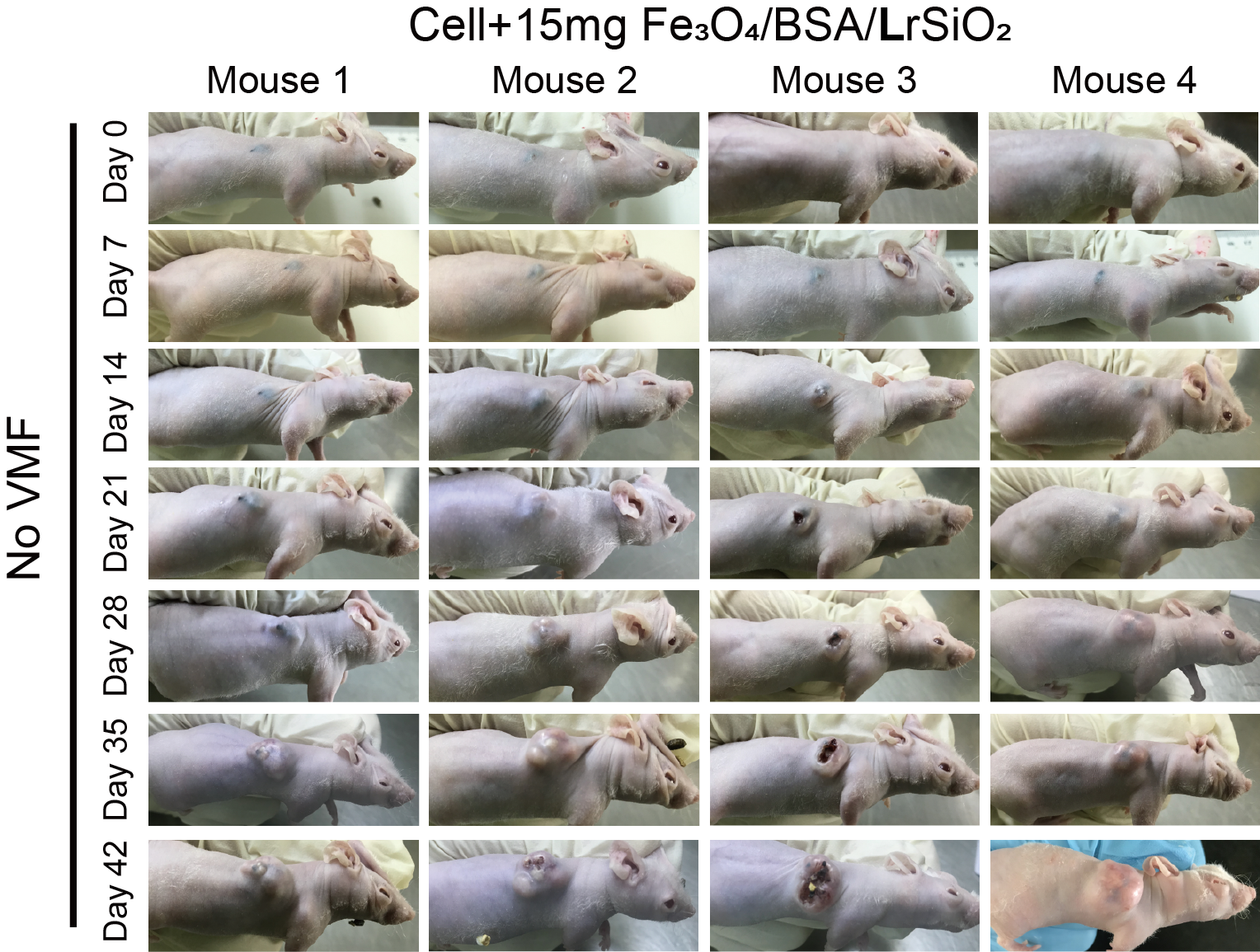


**Figure S10.** Tumor-bearing mice treated with 15 mg/mL Fe_3_O_4_/BSA/**L**rSiO_2_ (50 μL for each mouse)


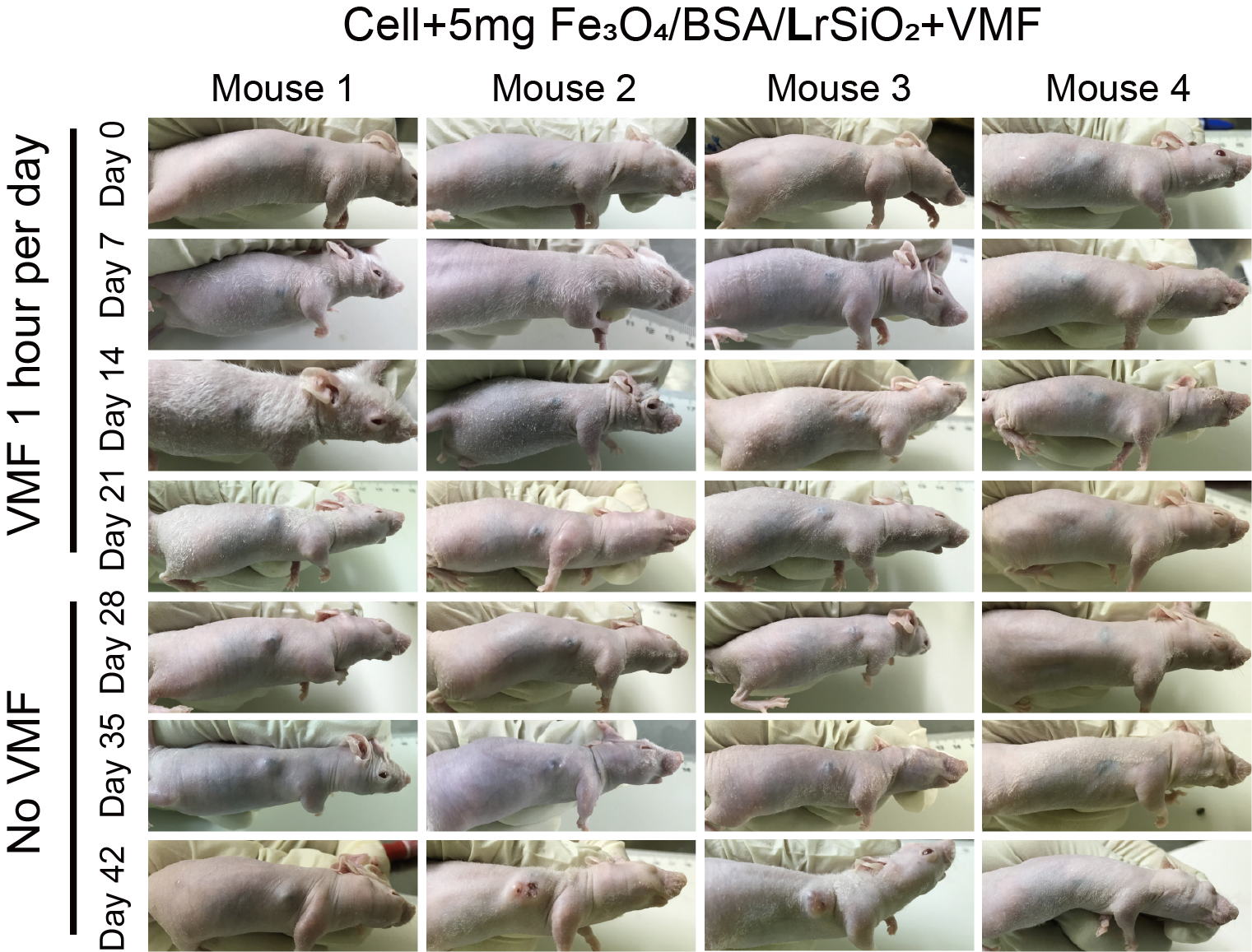


**Figure S11.** Tumor-bearing mice treated with 5 mg/mL Fe_3_O_4_/BSA/**L**rSiO_2_ (50 μL for each mouse) and VMF for 21 days


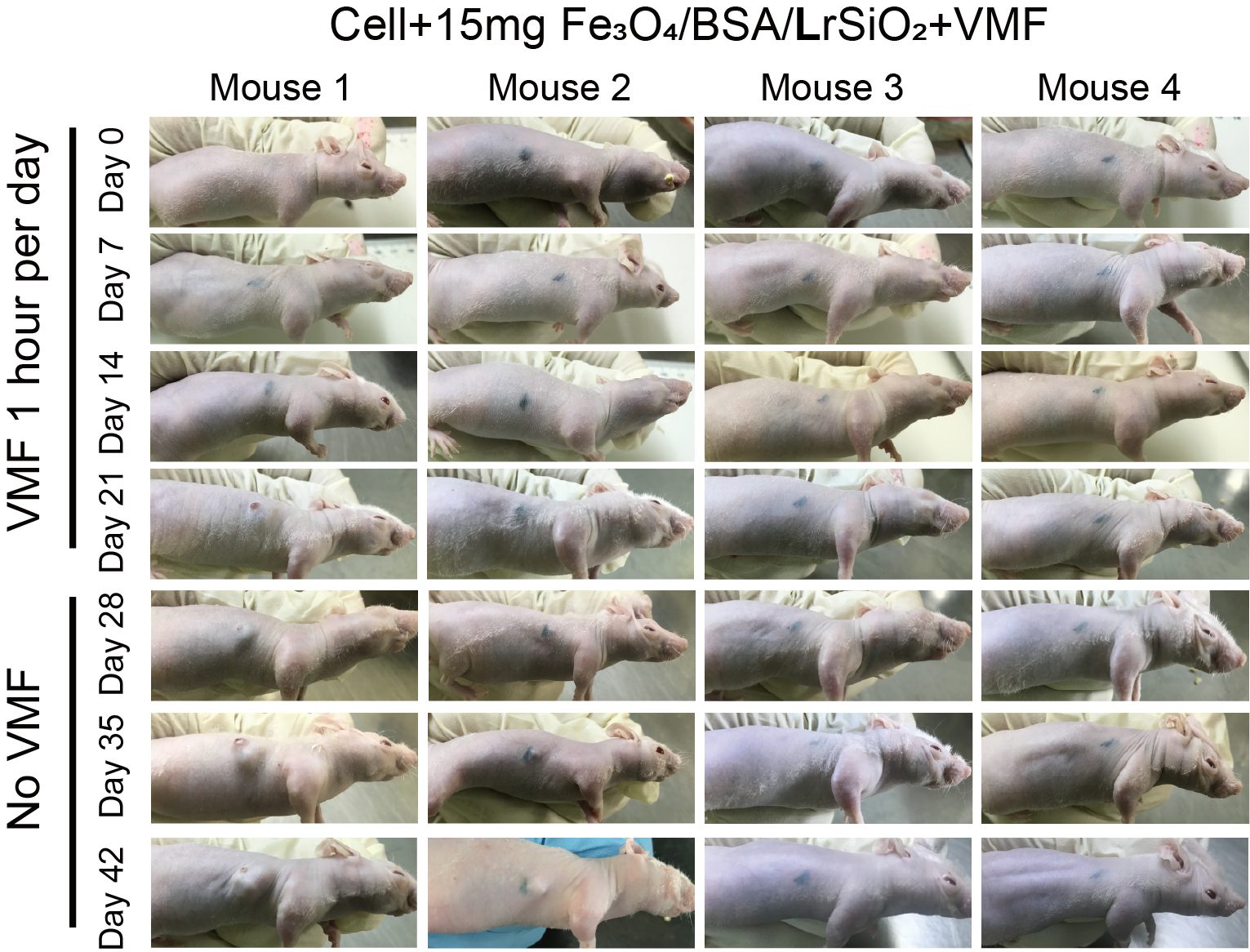


**Figure S12.** Tumor-bearing mice treated with 15 mg/mL Fe_3_O_4_/BSA/**L**rSiO_2_ (50 μL for each mouse) and VMF for 21 days
